# A Dual Approach Combining Metal‐Organic Frameworks and Commercial Solid‐Phase Microextraction Coatings for Monitoring Volatiles in Passion Fruit

**DOI:** 10.1002/jssc.70543

**Published:** 2026-09-27

**Authors:** Montse Saura‐Cayuela, Martina Rosso, Cecilia Cagliero, Jorge Pasán, Juan H. Ayala, Verónica Pino, María J. Trujillo‐Rodríguez

**Affiliations:** ^1^ Laboratorio de Materiales para Análisis Químico (MAT4LL) Departamento de Química Facultad de Ciencias Universidad de La Laguna (ULL) San Cristóbal de La Laguna Spain; ^2^ Unidad de Investigación de Bioanalítica y Medio Ambiente Instituto Universitario de Enfermedades Tropicales y Salud Pública de Canarias (IUETSPC) Universidad de La Laguna (ULL) San Cristóbal de La Laguna Spain; ^3^ Dipartimento di Scienza e Tecnologia del Farmaco Università di Torino Torino Italy; ^4^ Centro de Investigación Biomédica en Red de Enfermedades Infecciosas (Ciberinfec) Instituto de Salud Carlos III Madrid Spain

**Keywords:** gas chromatography‐mass spectrometry, metal‐organic frameworks, passion fruit, solid‐phase microextraction, volatiles

## Abstract

This study presents the applicability of a solid‐phase microextraction (SPME) coating based on the CIM‐80(*Al*) metal‐organic framework (MOF), prepared by in situ solvothermal growth onto braided silver wires, for the untargeted analysis of passion fruit juices. After the characterization of the MOF‐coated fiber by Fourier‐transform infrared spectroscopy, scanning electron microscopy, and powder X‐ray diffraction, it was applied in both headspace (HS)‐ and direct immersion (DI)‐ SPME in combination with gas chromatography‐mass spectrometry. The studies were carried out in comparison with three commercial SPME fibers. Considering all the fibers studied, up to 52 and 26 compounds were tentatively identified via HS‐ and DI‐SPME, respectively. Besides, all fibers presented adequate reproducibility and similar lifetime, even in the DI mode and despite the sample complexity. In HS‐SPME, a total of 25 compounds were tentatively identified with the MOF‐coated fiber. From these analytes, 10 compounds (∼42%) were exclusively detected with the MOF‐coated fiber. In DI‐SPME, 10 compounds were tentatively identified with the MOF‐coated fiber, and four of these compounds (∼50%) were exclusively detected with this fiber. Overall, the results indicate that the MOF‐coated fiber offers a superior selectivity for heavier, less volatile, and less polar analytes, therefore acting as a complementary tool for commercial coatings when intending metabolomic studies for food analysis. The dual approach with MOF‐coated fiber and commercial SPME coatings provides more comprehensive information for food analysis.

## Graphical abstract

A CIM‐80(*Al*) metal‐organic framework coating is proposed for the untargeted analysis of passion fruit juices using either headspace or direct immersion solid‐phase microextraction coupled with gas chromatography‐mass spectrometry. The CIM‐80(*Al*)‐coated fiber enabled the annotation of 14 unique compounds not detected with commercial fibers. The complementary use of both fiber types in a dual approach provides a powerful strategy for achieving more comprehensive chemical profiling in food analysis.

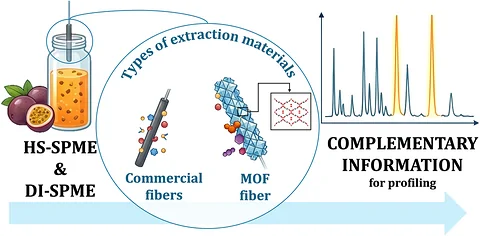

## 1 Introduction

Passion fruit (*Passiflora edulis* Sims.) is a tropical fruit highly valued for its intense and distinctive flavor, which plays a key role in its sensory appeal and industrial applications. The flavor of passion fruit is a combination of aromas and tastes from guava, mango, apple, banana, lychee, strawberry, lemon, and pineapple [1]. From a chemical perspective, flavor depends on a synergy between volatile organic compounds (VOCs) and non‐volatile organic compounds (NVOCs). NVOCs such as citric acid modulate the sour taste in the fruits, whereas soluble sugars contribute to sweetness [2]. The VOC profile of the fruit is dominated by esters, which are considered the main contributors to its fruity and sweet flavor. In addition, carotenoid‐derived C_13_ norisoprenoids like β‐ionone contribute floral and citrus notes. Other classes of volatiles, such as sulfur compounds, alcohols, aldehydes, and ketones, are also present and play an important role in providing green, fresh, and ripe sensory nuances [3, 4]. Therefore, understanding the volatile profile of passion fruit is the first step for describing its composition from a sensory perspective. In addition, the volatile profile can offer useful information for quality control, authenticity assessment, and varietal differentiation, aspects that also affect the final consumer acceptance of the fruit [5].

Solid‐phase microextraction (SPME) in the fiber format has unequivocally established itself as a key technique in contemporary food analysis, whether implemented in headspace (HS) or direct immersion (DI) modes, owing to its simplicity, solvent‐free nature, high sensitivity, and easy coupling with modern chromatographic systems [6, 7].

The success of in‐fiber SPME is linked to the nature of the sorbent coating, which is one of the most critical factors influencing the number and type of analytes that can be extracted, and its performance. In the analysis of fruit and fruit juices, commercial SPME coatings mostly include divinylbenzene/carboxen/polydimethylsiloxane (DVB/Car/PDMS), which is quite useful for the simultaneous determination of a wide range of polar/semipolar compounds [1, 8], although Car/PDMS has also shown utility for polar/semipolar VOCs with lower molecular weight and higher volatility [9, 10]. Several studies focused on elucidating the volatile profile of different passion fruit varieties [11, 12], studying ripening [13] and post‐ripening [14], and monitoring changes in the volatile profile during juice preservation [15, 16, 17] and fermentation [1]. By using DVB/Car/PDMS‐HS‐SPME and bidimensional gas chromatography coupled with time‐of‐flight mass spectrometry (GC × GC‐TOF MS), up to 134 VOCs were identified in fresh passion fruit (mostly categorized by esters, alcohols, and monoterpenes), and 102 VOCs were found in juices processed via thermal treatments (mostly esters, followed by alcohols) [17]. When implementing Car/PDMS‐HS‐SPME‐GC‐MS for monitoring 18 fruit juices from three passion fruit varieties, up to 135 VOCs were determined, mainly esters such as hexyl hexanoate and hexyl butanoate (HB), terpenes such as limonene, and alcohols such as 1‐hexanol and linalool (Lina) [12]. Apart from commercial fibers, many studies have reported alternative SPME coatings based on new materials such as carbon (nano)materials, alternative polymers, covalent organic frameworks, and metal‐organic frameworks (MOFs) [18, 19, 20]. Among them, MOFs have gained considerable attention, owing to their exceptionally high surface area, tunable porosity, and chemical selectivity. However, despite these properties make MOFs particularly attractive as coating materials, most of the reported applications focus on environmental analysis [20, 21, 22, 23, 24].

The use of SPME coatings based on novel materials for fruit and fruit juice analysis is more limited, most likely due to poor performance for minority compounds as a result of displacement effects in HS‐SPME [25, 26, 27], and/or fiber instability when using DI‐SPME. With respect to the use of MOFs as SPME coatings for this application, most of the studies use MOF/polymer composites rather than neat MOFs. Neat MOF coatings can offer higher thermal stability and thus lower fiber bleeding. An example of SPME composite for the analysis of orange, apple, and grape juices has been reported, using specifically polyacrylonitrile/Ni‐MOF coatings prepared via electrospinning [28]. At the same time, a study for the analysis of orange juice utilized a MOF‐on‐MOF system, particularly MIL‐88B‐on‐UiO‐66, that was glued onto a stainless‐steel support with a polyimide resin adhesive to generate the SPME fiber [29]. In addition, the applications developed with these hybrid MOF‐fibers were mostly focused on the targeted analysis of a relatively limited group of organic pollutants, including pesticides [28, 30, 31], polychlorinated biphenyls [29], or polycyclic aromatic hydrocarbons (PAHs) [32]. There are not studies with novel sorbents, including MOF sorbents, for monitoring phytochemicals or natural volatiles from fruit juices.

For untargeted volatile analysis using in‐fiber SPME‑GC‐MS, there are already well‑established methods, but so far these studies have only used conventional commercial fibers [12, 33] and not MOF‑based coatings, indicating a clear research opportunity to integrate MOF‑SPME with untargeted metabolomics workflows. At the same time, it is also important to note that these studies mainly focus on HS‐SPME, with few studies reporting the use of DI‐SPME [34].

This work proposes a neat MOF SPME coating onto a silver support, using specifically the CIM‐80(*Al*) MOF, with adequate robustness, as an alternative coating for analyzing passion fruit juice using both HS‐SPME and DI‐SPME coupled with GC and MS detection. The study is intended for monitoring not only the volatile profile but also the semi‐volatile profile of the passion fruit juice samples. Furthermore, there is a proper comparison of the performance of the MOF‐coated SPME fiber to that of successful commercial fibers, to evaluate possible complementarity among fibers to get the entire information in terms of food profiling, while proposing MOF coatings for certain target compounds that are difficult to determine with commercial coatings.

## 2 Materials and Methods

### 2.1 Chemical and Materials

All chemicals were of analytical quality, and all solvents were of high‐performance liquid chromatography (HPLC) grade. Sodium chloride, used to adjust the ionic strength, was supplied by Sigma‐Aldrich/Merck KGaA (Darmstadt, Germany). Ultrapure water was obtained from a Purelab Flex system (18.2 MΩ·cm, TOC 2 µg·L^−1^) from ELGA LabWater (High Wycombe, UK).

The following analytical standards of VOCs were purchased from Sigma‐Aldrich/Merck KGaA: ethyl butanoate (EB), butyl acetate (BA), (‐)–α–pinene (Pin), myrcene (Myr), hexyl acetate (HA), (R)–(+)–limonene (Limo), eucalyptol (Eu), γ–terpinene (Ter), Lina, and HB, all used in the optimization of the HS‐SPME‐GC‐MS method using the commercial SPME fiber. Table S1 of the Electronic Supplementary Material shows their structures and main physicochemical characteristics. Besides, 16 PAHs, namely naphthalene (Nap), acenaphthylene (Acy), acenaphthene (Ace), fluorene (Flu), anthracene (Ant), phenanthrene (Phe), fluoranthene (Flt), pyrene (Py), benzo[a]anthracene (BaAnt), chrysene (Chry), benzo[k]fluoranthene (BkFlt), benzo[b]fluoranthene (BzbFlt), benzo[a]pyrene (BaPy), dibenzo[a,h]anthracene (dBahAnt), benzo[g,h,i]perylene (BghiPe), and indeno[1,2,3‐cd]pyrene (Ind), were acquired from Sigma‐Aldrich/Merck and were used to evaluate the lifetime of the MOF‐coated SPME fibers. All the standards had a purity level higher than 97%. Thus, individual stock solutions were prepared at 2000 mg·L^−1^ in methanol (in the case of VOCs) and acetonitrile (in the case of PAHs), supplied by Honeywell Fluka (Seetze, Germany). All these solutions were stored in a freezer at ‐10°C and protected from light. From these stock solutions, an intermediate solution containing VOCs at 20 mg·L^−1^ was prepared by diluting the individual stock solutions in methanol, and another one containing 3 mg·L^−1^ with all 16 PAHs, in this case obtained by dilution with acetonitrile. For the calculation of the linear retention index (LRI), 2 µL of a standard mixture of *n*‐alkanes (C_8_–C_40_) (acquired from Sigma‐Aldrich/Merck) was injected into the GC–MS under the same analysis conditions as those used with samples.

For the preparation of the MOF‐coated SPME fibers, silver wires with a purity of 99.99%, an average diameter of 125 µm, and treated with temper annealing were acquired from Goodfellow & Components (Huntingdon, England). Other MOF precursors and reagents were: mercaptoacetic acid (98%), aluminum nitrate nonahydrate (98%), mesaconic acid (99%), urea (99%), ethanol, and ammonia solution (25%, *w/v*). All these reagents were obtained from Sigma‐Aldrich/Merck.

Solvothermal reactors of Teflon (15 mL) and stainless‐steel autoclaves from Parr Instrument Company (Moline, IL, USA) were used for the synthesis of the MOF on the fibers.

HS‐ and DI‐SPME experiments were carried out using 10 mL amber glass vials with silicone/PTFE screw caps, I.D. 18 mm, supplied by Supelco (Bellefonte, PA, USA). For agitation, a brand magnetic stirring bar with polytetrafluoroethylene (PTFE) was used, with a length of 15 mm and a bar diameter of 4.5 mm, also supplied by Sigma‐Aldrich/Merck. The extractions were carried out using a metallic block placed on a magnetic stirring hotplate from P Selecta (Barcelona, Spain).

The commercial SPME fibers Car/PDMS (50/30 µm), PDMS (100 µm), and DVB/Car/PDMS (50/30 µm) were acquired from Supelco, as well as blank SPME assemblies (24 Ga) to prepare the MOF‐coated fibers.

Around 25 different passion fruits at the ripe stage were sampled from plants of the same specie (*Passiflora edulis* Sims.) located in La Planta land in Güímar, Tenerife, Spain (28.316286, ‐16.384467). All fruits were initially cut; the pulp was then extracted from the mature fruits, and the fruit juice was obtained in a blender after filtration to remove the seeds. All the fruits were mixed to ensure that the volatile profile was the same in all cases, to point out the importance of selecting coatings to provide higher coverage of the volatile profile. Then, the juices were stored in 50 mL polypropylene Falcon tubes, supplied by Corning Inc. (Tamaulipas, Mexico), frozen in liquid nitrogen, and stored at ‐80°C until the moment of analysis. Before analysis, each Falcon tube was defrosted in the lab for 1 h in the fridge, with the Falcon wrapped in aluminum foil to avoid losses by photodegradation due to sunlight.

### 2.2 Instrumentation and Conditions

The phase identification of the CIM‐80(*Al)* SPME fiber was carried out by X‐ray powder diffraction (PXRD) using an Empyrean diffractometer (PANalytical, Netherlands). Scanning electron microscopy (SEM) analysis of the fibers was performed using an EVO 15 microscope (ZEISS, Germany) equipped with a 50 mm^2^ silicon drift X‐MAX detector (Oxford Instruments, Abingdon, UK), to evaluate the morphology of the coating and to estimate the thickness.

The determination of the volatile compounds was performed using a GC‐MS equipment (Agilent Technologies), consisting of a gas chromatograph (model 8860) coupled to a triple quadrupole MS detector (model 5977B). An HP‐5 MS capillary column (5% phenyl‐methylpolysiloxane) (30 m × 0.25 mm I.D., 0.25 µm film thickness) from Agilent Technologies was used. *Splitless* injections were performed, with helium as the carrier gas at a constant flow rate of 1.0 mL·min^−1^ with a septum purge flow of 3 mL·min^−1^. The inlet temperature was kept at the desorption temperature selected for each SPME fiber: the maximum recommended by the fabricant of the commercial coatings, and the maximum dictated by previous studies with the MOF‐coated fiber [22, 23]. The oven temperature gradient program started at 40°C for 2 min, then increased up to 120°C at 10°C·min^−1^, then up to 300°C at 5°C·min^−1^, and finally kept at 300°C for 3 min. The transfer line was kept at 280°C and the ion source operated at 230°C in electron impact mode (70 eV). The quadrupole was set at 150°C. SIM mode was used during optimization to ensure proper quantification of the target analytes studied.

For the qualitative analysis of passion fruits, the MS operated in Scan mode. The scan interval for mass spectral data acquisition was 1.0 s, and data were collected over a mass range (𝑚/𝑧) from 30 to 300 (with a Scan time of 350 ms).

### 2.3 Optimum Conditions of the Methods

#### 2.3.1 Preparation of MOF‐coated Fibers

The MOF‐coated SPME fiber coating was prepared according to the procedure of Negrín et al. in 2026 [23] following an in situ solvothermal growth approach. Briefly, silver wires of approximately ∼10 cm were submerged in a concentrated ammonia solution for 1 min. Subsequently, the wires were washed with abundant water and ethanol, respectively (this was repeated several times until the pH of the cleaning water was around 7). Then, fibers were immersed in a 0.58 mM mercaptoacetic acid solution for 24 h to promote the thiol‐silver bond formation, leading to a surface functionalization with carboxylic groups for further in situ growth of the MOF. For each SPME fiber, two wires were assembled together and braided to increase the surface area exposed [23, 35]. Figure S1 shows the braiding method, which was previously reported [23].

The CIM‐80(*Al*)‐coated fibers were synthesized through a solvothermal method in a Teflon‐lined reactor, introducing 20 fibers per batch in 15 mL of a water solution containing the precursors of the MOF: 1 mmol of Al(NO_3_)_3_·9H_2_O (375 mg), 1 mmol of mesaconic acid (130 mg), and 0.5 mmol of urea (30 mg). The container was sealed in a stainless‐steel autoclave and placed in an oven at 150°C for 3 h.

The resulting MOF‐coated fibers were collected and carefully washed three times with water to remove any unreacted precursors from the pores, followed by washing with ethanol to replace the reaction solvent. The fibers were mounted onto in‐fiber SPME blank assemblies and were subjected to thermal activation at 280°C for 30 min.

#### 2.3.2 HS‐ and DI‐SPME‐GC‐MS

HS‐SPME extractions were performed using 5 mL of defrosted fruit juice, in which the NaCl content was adjusted to 15% (*w/v*). All fibers were exposed to the HS of the vial at 62°C under stirring at 500 rpm for 60 min (optimum conditions for Car/PDMS).

For the DI‐SPME procedure, 8 mL of defrosted fruit juice was used, with the fiber being immersed in the solution (the fruit juice) under the same conditions mentioned above, with the exception that no salt was added, and the extraction time was fixed to 60 min. In this mode, the fiber was immersed in ultrapure water for 1 s before GC desorption, to ensure removal of any sugars from the juice that could affect the fiber integrity when exposed to the high temperatures of the GC inlet.

In both in‐fiber SPME modes (HS‐ and DI‐), the desorption was accomplished in the GC inlet for 10 min at different temperatures depending on the fiber: 270°C for DVB/Car/PDMS, 280°C for PDMS and for the MOF‐coated fiber, and 310°C for Car/PDMS. All the fibers were conditioned at their corresponding temperature in the GC injector for 15 min every day prior to use.

To evaluate the lifetime and proper functioning of both the MOF braided SPME fiber and the commercial fibers in DI‐SPME of a complex sample, HS‐SPME of aqueous standards of 16 PAHs was performed in triplicate before and after the analysis of the passion fruit juice sample via DI‐SPME. The extraction procedure for the determination of the PAHs follows previous studies of the group [23]. Briefly, HS‐SPME is performed from 5 mL of aqueous standard solution of PAHs at 10 µg·L^−1^ in 20% NaCl (*w/v*) at 53°C for 57 min, and with 500 rpm agitation. The fiber was then subjected to desorption in the GC inlet for 10 min at the maximum working temperature for each fiber. Table S2 summarizes the conditions employed in each type of experiment.

The absence of *carryover* was ensured in all experiments by performing successive desorption steps in the GC‐MS and via blank extractions, using 20% (*w/v*) NaCl aqueous solution in HS‐SPME, and ultrapure water in DI‐SPME.

#### 2.3.3 Data Processing and Compound Identification

For data preprocessing, chromatograms were integrated using Agilent's Unknown Analysis (version 10.1) and Qualitative Analysis (version 10.0) software by deconvolution rather than by peak area, to emphasize the number of compounds rather than their abundance. A first peak list with retention time (RT), experimental LRIs, and MS data was obtained. Blank subtraction of a chromatogram related to the extraction/desorption of 20% (*w/v*) NaCl aqueous solution (in HS‐SPME) or ultrapure water (in DI‐SPME) was performed. LRIs were determined via the method introduced by Van den Dool and Kratz [36] for temperature‐programmed GC analysis.

For VOC identification, the MS data were compared with data from the NIST MS library (v. 2014), only considering the peaks that were present in the three replicates, R. match factors >70%, and a trapezoidal RT penalty function with an RT range of 9 s. The hits that meet these criteria were compared with theoretical LRIs, admitting ± 40 units as maximum tolerance between the experimental and literature LRIs. For attempt identification, the best hit was selected based on the highest R. match factor (identification level 2). In addition, confirmation was ensured for common VOCs by the injection of the corresponding standards in the GC‐MS (identification level 1) [37].

## 3 Results and Discussion

### 3.1 Characterization of the CIM‐80(*Al*) SPME fiber

The adequate synthesis of the MOF CIM‐80(*Al*) on the surface of the silver fibers was evaluated using Fourier‐transform infrared spectroscopy (FTIR), SEM, and PXRD. The SEM images of the MOF‐braided fibers show the presence of reproducible patches of large crystals with a size of around 1–3 µm (Figure S2‐A) sitting on top of a homogeneous layer of much smaller crystallites, as reported before [23]. Figure S2‐B shows the FTIR spectra measured directly on the CIM‐80(*Al*)‐coated fiber and that of the CIM‐80(*Al)* powder obtained during the synthetic procedure. All peaks corresponding to the symmetric and asymmetric stretching vibrations of the carboxylate groups (COO^−^) at 1600 and 1400 cm^−^^1^, as well as the C–O and Al–O vibrations at 1000 and 800 cm^−^^1^, respectively, of CIM‐80(*Al*) are also observed on the fiber support, confirming the correct formation of the MOF. Finally, the good agreement among the PXRD patterns of CIM‐80(*Al*)‐coated SPME fiber, the MOF powder obtained during the synthesis, and the simulated pattern from the crystal structure supports the formation of CIM‐80(*Al*) onto the fiber (Figure S2‐C). The characterizations provide evidence that the CIM‐80(*Al*) crystals have grown onto the metallic support, and these MOF‐coated fibers were utilized in this study, comparing their performance with that of commercial SPME fibers.

To ensure the reproducibility of the synthesis of the fibers, HS‐SPME‐GC‐MS analysis of an aqueous standard solution of PAHs (10 µg·L^−1^) (*n* = 3) was performed for each braided fiber synthesized. Figure S3 shows the results obtained with four different CIM‐80(*Al*) braided fibers after each synthesis, under optimum conditions and in triplicate [23]. Table S3 shows the intra‐batch RSD values obtained, between 1.05% and 14.7%, with the inter‐batch values between 5.55% and 18.4%, thus showing the adequacy.

### 3.2 HS‐SPME‐GC‐MS Method

The HS‐SPME‐GC‐MS method was selected, and the commercial SPME fibers were used at their optimum conditions. In all cases, the extractions were carried out under constant magnetic stirring at 500 rpm to ensure adequate homogenization.

Thus, in the case of the commercial Car/PDMS fiber, the optimum conditions were those optimized in a previous study [12]. Briefly, they involved 60 min for the extraction, 62°C for the extraction temperature, and 15% (*w/v*) for the NaCl content to ensure sufficient ionic strength to favor the salting‐out effect. The desorption conditions involved 310°C at the GC injection port for 10 min.

For the commercial DVB/Car/PDMS fiber, optimization focused on monitoring a group of target VOCs commonly found in fruit juice samples (shown in Table S1) using a Doehlert design. The variables considered in this experimental design were the extraction time (from 30 min to 60 min), the extraction temperature (from 30°C to 70°C), and the ionic strength (from 0% to 20% (*w/v*) NaCl content) (see Table S4). The reasons for selecting these minimum and maximum values relate to: (i) avoiding tedious protocols (long extraction times), (ii) minimizing the risks of volatilization of certain compounds (at high temperatures), and (iii) limiting the range for the ionic strength (to verify the influence of the salting‐out effect). The desorption temperature and desorption time were fixed to 270°C and 10 min, respectively. The desorption temperature is the maximum allowed for the fiber, and longer times are avoided during desorption to maximize the fiber lifetime.

The results obtained for the experiments were best fitted by second‐order polynomial functions, and the values of the constants and coefficients obtained are listed in Table S5. The coefficients of determination ranged between 61% and 89% (for a confidence level of 95%), indicating good correlation between the experimental data and the statistical model. The optimal conditions obtained for each target analyte by HS‐SPME using this DVB/Car/PDMS SPME fiber are shown in Table 1, using the response surfaces of the desirability function to select the optimal compromise conditions (Figure S4). In view of these results, the optimal compromise conditions were 60 min for the extraction time, 60°C for the extraction temperature, and 20% (*w/v*) for the NaCl content.

**TABLE 1:** Optimum values and compromising conditions obtained for each target analyte, using the Doehlert design and HS‐SPME‐GC‐MS (SIM) for DVB/Car/PDMS.

Analyte	Optimum extraction time (min)	Optimum extraction temperature (°C)	Optimum NaCl content (%, *w/v*)	Response, as peak area ×10^6^ (counts)	
				Optimum for each value	Selected conditions
EB	62.3	67.3	24.2	3.75	2.79
BA	27.7	32.7	24.9	2.08	1.37
Pin	62.3	32.7	4.10	10.2	8.08
Myr	62.3	67.3	3.49	13.6	10.1
HA	62.3	67.3	13.7	20.4	17.6
Limo	62.3	67.3	14.9	17.8	15.9
Eu	62.3	46.8	25.0	11.4	9.66
Ter	62.2	32.7	4.36	28.1	18.8
Lina	62.3	55.2	25.0	14.7	12.8
HB	62.1	67.3	16.3	23.8	20.4
Selected conditions					
	*60*	*60*	*20*	—	—

Both commercial SPME fibers, Car/PDMS and DVB/Car/PDMS, were compared under their optimal extraction conditions and in triplicate, monitoring the selected VOCs (as shown in Figure 1). Both fibers exhibit quite similar results; some target compounds are slightly favored by Car/PDMS (EB, BA, Myr, HA, Limo, and Eu), and the remaining VOCs by the DVB/Car/PDMS (Pin, Ter, Lina, and HB). Therefore, the HS profiles were obtained with both commercial fibers to avoid loss of information.

**FIGURE 1:**
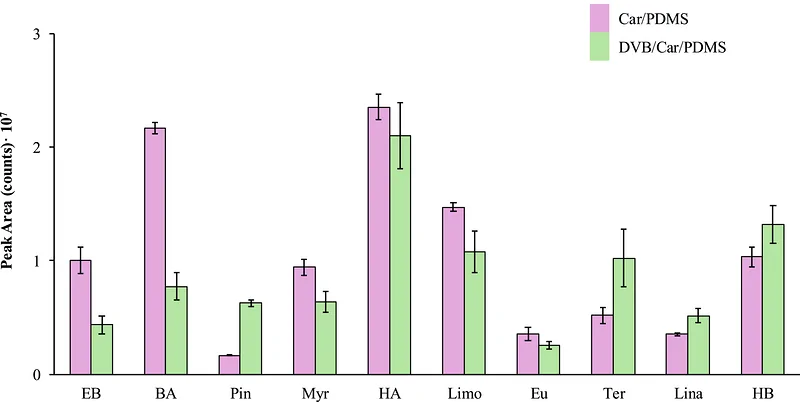
HS‐SPME‐GC‐MS results, monitored as peak areas, obtained with each commercial SPME fiber under optimal extraction conditions for an aqueous solution of 100 µg·L^−1^: Car/PDMS (62°C, 60 min, and 15% NaCl) and DVB/Car/PDMS (60°C, 60 min, and 20% NaCl) and in triplicate.

The CIM‐80(*Al)*‐coated braid fiber was evaluated under the two optimum conditions of commercial fibers (see Figure S5A). Highly volatile aroma compounds were poorly extracted by the CIM‐80(*Al*)‐coated fiber, showing lower signal intensities compared to the commercial fibers. Despite its lower performance toward the selected group of targeted VOCs, the MOF fiber showed higher peak areas for esters than other groups of compounds. In fact, when using the CIM‐80(*Al*)‐coated fiber at the optimum conditions of DVB/Car/PDMS, the peak areas of EB, BA, and HB were 14–440 times higher than the peak areas of the remaining VOCs. In view of these results, the MOF fiber was tested in the following HS experiments with the same optimum conditions obtained for DVB/Car/PDMS (60 min, 60°C, and 20% NaCl, as shown in Table S2).

### 3.3 HS Profile of Passion Fruits

The volatile profiles of passion fruit were obtained via HS‐SPME‐GC‐MS, selecting in this case an untargeted approach, using the three fibers studied (Car/PDMS, DVB/Car/PDMS, and CIM‐80(*Al*)), the optimal conditions for each fiber (Table S2), and performing experiments in triplicate. The MOF fiber exhibits a higher response for heavier compounds in the volatile profiles obtained for each SPME fiber (Figure 2).

**FIGURE 2:**
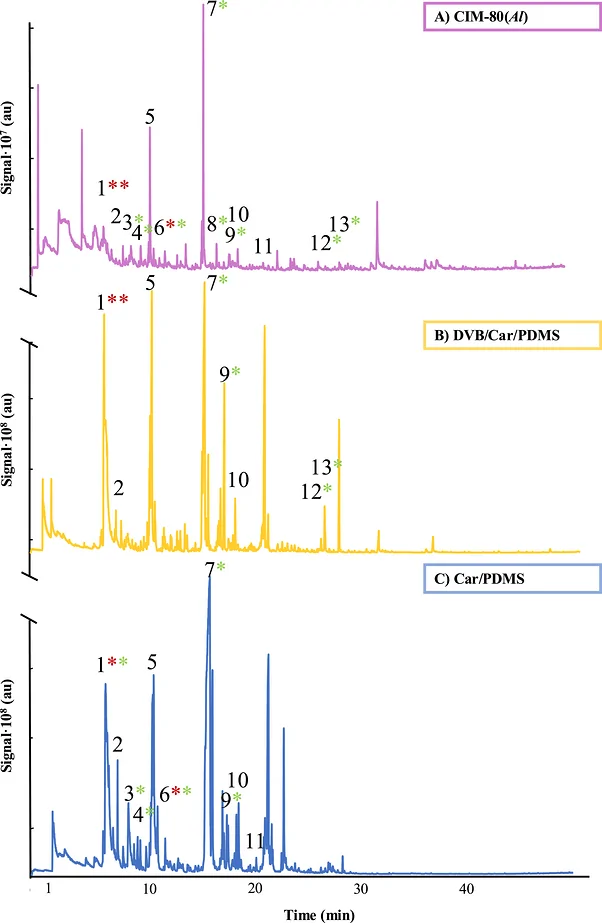
Representative chromatograms in scan mode obtained from 5 mL of passion fruit juice by HS‐SPME‐GC‐MS using each fiber under optimum conditions: Car/PDMS (62°C, 60 min, and 15% NaCl), DVB/Car/PDMS (60°C, 60 min, and 20% NaCl), and CIM‐80(*Al*) (60°C, 60 min, and 20% NaCl), in triplicate. Analytes identified in the passion fruit samples analyzed: 1) EB, 2) ethyl hexanoate, 3) HA, 4) β−ocimene, 5) alloocimene, 6) HB, 7) (Z)‐geranylacetone, 8) γ‐heptyl butyrolactone, 9) hexyl hexanoate, 10) dihydroactinidiolide, 11) 3‐mercaptohexyl hexanoate, 12) hexadecanoic acid, and 13) octadecanoic acid. *Compounds preselected in the optimization of the HS‐SPME‐GC‐MS method. *Compounds selected in the SIM mode study.

In total, 52 different compounds have been tentatively identified with all fibers, with six compounds detected with all fibers: three esters (EB, ethyl hexanoate, and hexyl hexanoate), three terpenes (alloocimene, (Z)‐geranylacetone, and dihydroactinidiolide). This aspect points out the different selectivity of the fibers for the analytes despite analyzing the same sample (see Figure 3A and Figure S6). The 52 VOCs mainly comprise esters (29 compounds, representing ∼59% of the identified profile), terpenes (15 compounds, ∼27%), and acids (three compounds, ∼6%). The MOF‐coated fiber identified a wider range of compound families than the commercial fibers (Figure S6).

**FIGURE 3:**
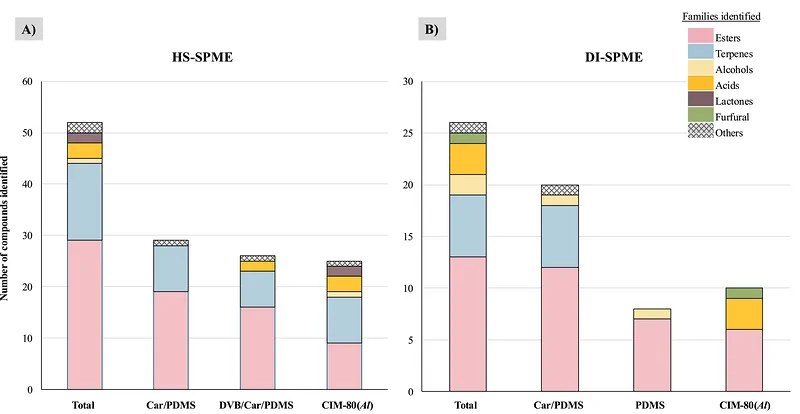
Families of compounds identified using the HS‐ and DI‐SPME‐GC‐MS methods with different fibers under optimum conditions.

In terms of the overall number of compounds identified, the Car/PDMS fiber permitted the identification of 29 compounds, the DVB/Car/PDMS identified 26 compounds, whereas the CIM‐80(*Al*) fiber identified 25 compounds, as shown in Table S6. Five target VOCs (specifically EB, HA, Pin, HB, and Limo), initially used in the optimization, were also identified in the passion fruit sample. The synergistic presence of these compounds generates a fruity and floral aroma. Ethyl butanoate and γ‐dodecalactone produce a fruity aroma that has been detected in passion fruit, apricot, and tropical fruits [38, 39]. Terpenes produce a more floral aroma, and both have been previously reported in tropical fruits such as pineapple [40].

Ten compounds were detected and identified only with the MOF‐coated fiber. These VOCs comprise four terpenes, one ester, one alcohol, two lactones, one acid, and one ketone. Among the terpenes, it is interesting to highlight the detection of Ter. This monoterpene has been reported in *Passiflora edulis*, although its presence is not universal across all species [11]. Another terpene only identified with the MOF fiber was pseudoionone, a compound with a balsamic, citrus, and fruity scent [40]. In addition, pseudoionone is considered a biosynthetic precursor of β−ionone; it is a key odor compound in passion fruit and was identified with both DVB/Car/PDMS and the MOF fiber. Thus, the use of the MOF fiber is crucial for understanding the metabolic biosynthesis of ionones. Besides, this study identified one ester, butyl‐2‐butenoate, and one alcohol (benzyl alcohol), which are known for their contribution to floral notes in fruits [32]. Furthermore, two identified lactones were γ‐dodecalactone and γ‐heptyl butyrolactone, both contributing to a peach and fruity flavor.

The results obtained have been compared with previous studies in the literature that also focused on HS‐SPME (Table S7). Several studies have provided a similar number of identified volatiles, from 31 VOCs [13] to 78 VOCs [41] depending on the study, and mostly when using DVB/Car/PDMS‐HS‐SPME‐GC‐MS. There are other studies that have reported a higher number of compounds than this study, but either used more powerful separation and detection techniques such as GC × GC‐TOF MS [17] or used identification protocols that were not based on LRI identity confirmation [14, 42]. Other studies reported a higher number of compounds analyzed in several fruit juices independently [11, 12]. In comparison, in this study, a pool of fruit juices was analyzed to ensure the same sample composition in all the experiments.

In summary, this MOF SPME coating is highly advantageous for untargeted analysis, as it can detect compounds that commercial fibers cannot capture under their optimum conditions. Therefore, these new extraction phases in HS‐SPME‐GC‐MS will allow for a much broader study of the volatile profile of passion fruit juice (and other food matrices), as they can give a richer profile; thus, they can serve as a complementary tool to properly define the presence of semi‐volatile compounds in food samples.

To gain insights into the results obtained, nine compounds were selected. Their selection was based on two criteria: (i) analytes identified in passion fruit independently of the type of SPME fiber, and (ii) analytes with different volatility and molecular weight, in order to have a broader picture of behavior. These compounds were: ethyl butanoate (EB), hexyl acetate (HA), β‐ocimene, hexyl butanoate (HB), hexyl hexanoate, (Z)‐geranylacetone, hexadecanoic acid, γ‐heptyl butyrolactone, and octadecanoic acid. These compounds were monitored in SIM mode to semi‐quantitate their presence in passion fruit juice (see Table S8). Furthermore, as there were no important differences for these compounds between commercial Car/DMS and DVB/Car/PDMS fibers, this study was conducted with Car/PDMS. Figure S7 shows the peak areas after application of the optimum HS‐SPME‐GC‐MS method with Car/PDMS and the MOF‐coated fiber at conditions depicted in Table S2, with experiments performed in triplicate. Based on these results, there is greater compatibility between the commercial fiber and polar and low molecular weight analytes, but as the molecular weight and non‐polarity of the analytes increase, there is a clear trend of favored extraction with the MOF fiber. In fact, there is a 111–302‐fold increase in the peak area for the less polar VOCs (i.e., hexadecanoic acid, γ‐heptyl butyrolactone, and octadecanoic acid) when using CIM‐80(*Al*)‐coated fiber. In addition, as occurred in Section 3.2, the highest peak areas were obtained for esters like HB and hexyl hexanoate when using the MOF‐coated fiber.

Trying to evaluate whether MOF‐coated fibers are more suitable in HS‐SPME to monitor those difficult compounds with high molecular weight and relatively lower polarity, the PDMS fiber was used for the same experiments. In general, PDMS is not used in food analysis because it is a non‐polar stationary phase (as is the behavior of the MOF fibers in this application). The comparison was performed under the same extraction conditions optimized in this study (Table S2). It was found that, although the PDMS fiber has a greater affinity for non‐polar compounds, it was unable to identify any of the selected compounds in SIM mode. In other words, the MOF‐coated fiber demonstrates superior performance in HS‐SPME‐GC‐MS for less‐polar and high molecular weight compounds in fruit matrices. This could be linked to the adsorption‐like mechanism of this MOF‐coated fiber [23] rather than the absorption behavior of PDMS.

### 3.4 DI‐SPME‐GC‐MS Method

The composition of the less volatile compounds present in passion fruit juice was investigated using DI‐SPME‐GC‐MS. In this study, the fruit juice (without salt addition) was directly analyzed by DI of the fibers. The sample volume was fixed to 8 mL of juice to ensure the vial was filled with sample while preventing contact with the septum, and the stirring rate and extraction time were fixed using those optimized for HS‐SPME. The extraction time was evaluated between 20 and 120 min, with no significant variation in the obtained volatile profile. Therefore, an extraction time of 60 min was selected. This time corresponds to the duration of the GC‐MS chromatogram, and thus, it does not affect the sample throughput. Desorption conditions were the same as those used in HS mode (10 min and desorption temperature depending on the fiber; see Section 2.3.3), and GC‐MS operated in Scan mode for obtaining the DI profile (see Table S2 with the rest of the conditions).

Figure 4 shows the difference in the profiles obtained when using three fibers (Car/PDMS, PDMS, and CIM‐80(*Al*)) and the same type of sample (*n* = 3). A total of 26 compounds were tentatively identified with the three fibers, of which 13 were esters, six terpenes, three acids, two alcohols, one furfural, and a norisoprenoid (theaspirane, see Table S9). Six of the 13 esters were detected with the three fibers: EB, HB, ethyl octanoate, 3‐hexen‐1‐yl hexanoate, hexyl hexanoate, and hexyl octanoate. All these compounds were also previously identified in HS in this study.

**FIGURE 4:**
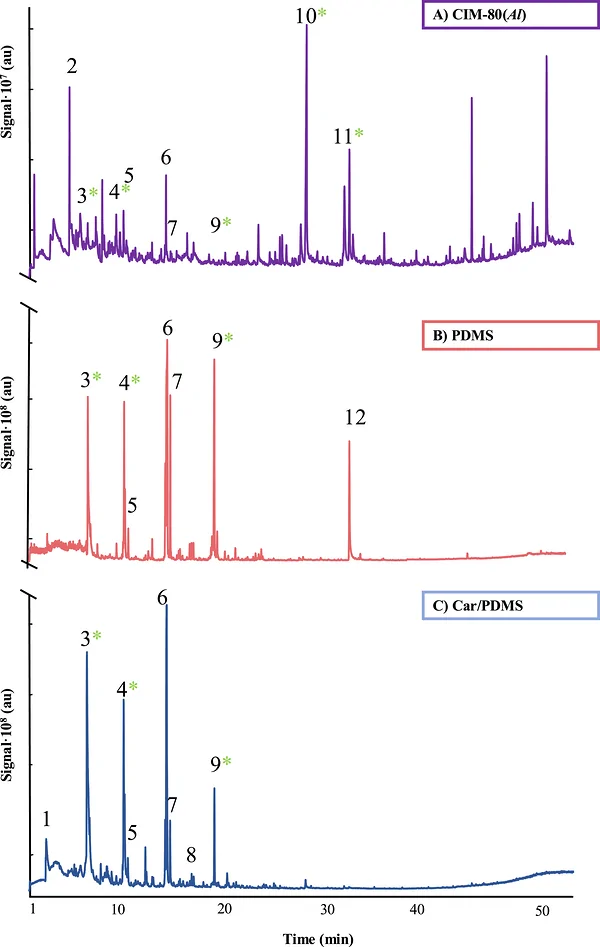
Representative chromatograms obtained from 8 mL of passion fruit juice by DI‐SPME‐GC‐MS with each fiber under optimum conditions: Car/PDMS (62°C and 60 min), PDMS (60°C and 60 min), and CIM‐80 (*Al*) (60°C and 60 min), in triplicate. Analytes identified in the passion fruit samples analyzed: 1) EB, 2) 5‐methylfurfural, 3) ethyl hexanoate, 4) HB, 5) ethyl octanoate, 6) 3‐hexenyl hexanoate, 7) hexyl hexanoate, 8) ionone, 9) hexyl octanoate, 10) hexadecanoic acid, 11) octadecanoic acid, and 12) (Z)‐geranylgeraniol *Compounds selected in the SIM mode study.

Fourteen compounds were exclusively identified with the Car/PDMS fiber, of which six were terpenes, six esters, one alcohol, and theaspirane. The PDMS fiber was able to detect a total of eight compounds. From these, two compounds could not be identified with the other fibers: octyl hexanoate and geranylgeraniol. Meanwhile, the MOF‐coated fiber was able to identify 10 compounds in total, and four of these compounds could not be determined with the remaining fibers. These four compounds included three acids and one furfural. The MOF‐coated fiber shows a preference for acids such as hexadecanoic acid, 9‐hexadecanoic acid, and octadecanoic acid. 5‐Methylfurfural, which was identified only when using the MOF‐coated fiber, is a compound derived from sugar and biomaterials, with a taste description of cherry and other fruits, almond and other nuts, cocoa, coffee, and amaretto‐type flavors [40].

Five compounds were selected in SIM mode to explore the interaction of these compounds with the fibers. Two esters with high volatility and low molecular weight were included in this selection: ethyl hexanoate and HB. Besides a compound with lower volatility, hexyl octanoate, and two compounds with high molecular weight, hexadecanoic acid and octadecanoic acid, were also selected (see Table S10). In this SIM study, different extraction times were studied, but no differences were observed between 20 min and 2 h of extraction. Therefore, 60 min of extraction was chosen as the time for the comparative study, and the obtained results are shown in Figure S8. The observed behavior resembled that of HS‐SPME, where compounds with higher volatility and lower molecular weight, such as EH and HB, were favorably extracted by the Car/PDMS fiber, while the MOF‐coated fiber showed higher affinity for molecules with higher molecular weight, yielding results even better than those of the commercial PDMS fiber.

It is also important to evaluate the stability of the MOF‐coated fibers (comparing it with that of commercial fibers) when used in DI mode with complex samples such as fruit juices. Such reuse was evaluated after its application by DI in juice samples. To this end, the set of experiments were as follows: 1) HS‐SPME‐GC‐MS analysis of an aqueous standard solution of PAHs (10 µg·L^−1^) (*n* = 3), 2) DI‐SPME‐GC‐MS analysis of a juice sample (*n* = 1), and 3) HS‐SPME‐GC‐MS analysis of an aqueous standard solution of PAHs (10 µg·L^−1^) (*n* = 3) (the detailed conditions are described in Table S2). Figure S9 shows the results obtained for Car/PDMS and the CIM‐80(*Al*) fiber. The extraction efficiency for PAHs with both the commercial and the MOF‐coated fibers was, on average, similar before and after the DI procedure. In addition, the fibers were able to resist more than 20 consecutive DI‐SPME analyses (together with the corresponding HS‐SPME extractions performed before and after DI‐SPME to ensure no damage to the fibers), indicating that there is not important sorbent loss or damage after exposing the fibers directly to the juice. This supports the robustness of the SPME coatings despite being immersed in complex juice samples.

## 4 Conclusions

This work has extended the use of MOF SPME coatings for the analysis of passion fruit in both HS‐ and DI‐SPME‐GC‐MS incorporating an untargeted analysis approach. The CIM‐80(*Al*) coating used in this study demonstrated different selectivity with respect to commercial fibers. Indeed, the MOF fiber enabled the tentative identification of 14 compounds that were not identified with conventional fibers: 10 compounds with HS‐SPME and four compounds with DI‐SPME. Although the commercial Car/PDMS and DVB/Car/PDMS were able to identify a higher number of compounds in either HS‐ and/or DI‐, the MOF fiber showed enhanced response toward heavier and less volatile analytes, allowing the identification of several compounds that had not been previously reported in passion fruit studies. The fiber also showed better performance than PDMS in terms of the number of volatiles. These results highlight the ability of MOF coatings to extract compounds that are more difficult to detect using conventional SPME fibers.

The CIM‐80(*Al*)‐coated SPME fibers also showed similar reproducibility and lifetime (around 20 analyses in total in DI‐SPME) with respect to commercial fibers, even when using DI‐SPME with non‐diluted passion fruit juice.

The results indicate that MOF‐coated SPME coatings constitute a promising alternative for the characterization of complex food matrices. Their different selectivity and affinity for heavier and less volatile analytes make them a valuable complementary tool to commercial fibers, permitting the obtaining of a more comprehensive and less biased volatile profile in passion fruit juice and, by extension, in other food samples, when using this dual approach. Thus, the combination of MOF and commercial SPME fibers constitutes a simple strategy for untargeted metabolomic studies of food, permitting a more holistic overview.

## Author Contributions

**Montse Saura‐Cayuela**: data curation; formal analysis; investigation; methodology; writing – original draft preparation; validation; visualization. **Martina Rosso**: data curation; formal analysis; investigation. **Cecilia Cagliero**: formal analysis; validation; writing – review & editing. **Jorge Pasán**: conceptualization; formal analysis; funding acquisition; writing – review & editing; validation. **Juan H. Ayala**: software; formal analysis; validation. **Verónica Pino**: conceptualization; formal analysis; funding acquisition; methodology; project administration; supervision; writing – review & editing; validation. **María J. Trujillo‐Rodríguez**: conceptualization; formal analysis; investigation; funding acquisition; methodology; supervision; validation; writing – review & editing; visualization.

## Conflicts of Interest

The authors declare no conflicts of interest.

## Funding

This work was developed within the scope of the project ref. PID2023‐147246OB‐I00 funded by MICIU/AEI/10.13039/501100011033/ERDF, EU. Montse Saura‐Cayuela thanks her for her FPI predoctoral contract at Universidad de La Laguna, co‐financed by Agencia Canaria de Investigación, Innovación y Sociedad de la Información (ACIISI) de la Consejería de Universidades, Ciencia e Innovación y Cultura, and by the European Social Fund Plus (ESF+) integrated operation program of the Canary Islands 2021‐­ 2027, Axis 3 Topic Priority 74 (85%). Martina Rosso thanks the Erasmus+ program for her stay at Universidad de La Laguna. María J. Trujillo‐Rodríguez thanks her Ramón y Cajal contract (ref. RYC2021‐032502‐I) at Universidad de La Laguna (ULL), contract with funding of MICIU/AEI/10.13039/501100011033 – previously MICIN—and the European Union «NextGenerationEU»/PRTR; and both MICIU/AEI and ULL for the additional funding related to the contract.

## Supporting information




**Supporting Information**: jssc70543‐sup‐0001‐SuppMat.pdf

## Data Availability

The data that support the findings of this study are available from the corresponding author upon reasonable request.
