# Supplementary material for: A Dual Approach Combining Metal‐Organic Frameworks and Commercial Solid‐Phase Microextraction Coatings for Monitoring Volatiles in Passion Fruit
Source: J Sep Sci. 2026 Sep 27;49(9):e70543. doi: 10.1002/jssc.70543 (PMC13616997; doi:10.1002/jssc.70543)
Supplement: Supplementary file 1 [file JSSC-49-e70543-s001.pdf]

Electronic Supplementary Material

A dual approach combining metal-organic frameworks- and commercial- solid-phase microextraction coatings for monitoring volatiles in passion fruit

Montse Saura-Cayuela[1,2], Martina Rosso[1,3], Cecilia Cagliero[3], Jorge Pasán[1], Juan H. Ayala[1], Verónica Pino[1,2,4], María J. Trujillo-Rodríguez[1,2,*]

[1]*Laboratorio de Materiales para Análisis Químico (MAT4LL), Departamento de Química, Facultad de Ciencias, Universidad de La Laguna (ULL), 38206 San Cristóbal de La Laguna, Spain*

[2]*Unidad de Investigación de Bioanalítica y Medio Ambiente, Instituto Universitario de Enfermedades Tropicales y Salud Pública de Canarias (IUETSPC), Universidad de La Laguna (ULL), 38206 San Cristóbal de La Laguna, Spain*

[3]*Dipartimento di Scienza e Tecnologia del Farmaco, Università di Torino, 10125 Torino, Italy*

[4]*Centro de Investigación Biomédica en Red de Enfermedades Infecciosas (Ciberinfec), Instituto de Salud Carlos III, 28029 Madrid, Spain*

INDEX

FIGURES

Figure S1 2
Figure S2 3
Figure S3 4
Figure S4 5
Figure S5 6
Figure S6 7
Figure S7 8
Figure S8 9
Figure S9 10

TABLES

Table S1 11
Table S2 13
Table S3 14
Table S4 15
Table S5 16
Table S6 17
Table S7 20
Table S8 22
Table S9 23
Table S10 26

Figure S1. Scheme showing the braiding procedure with the silver wires (already etched) used as solid support for the SPME fibers.

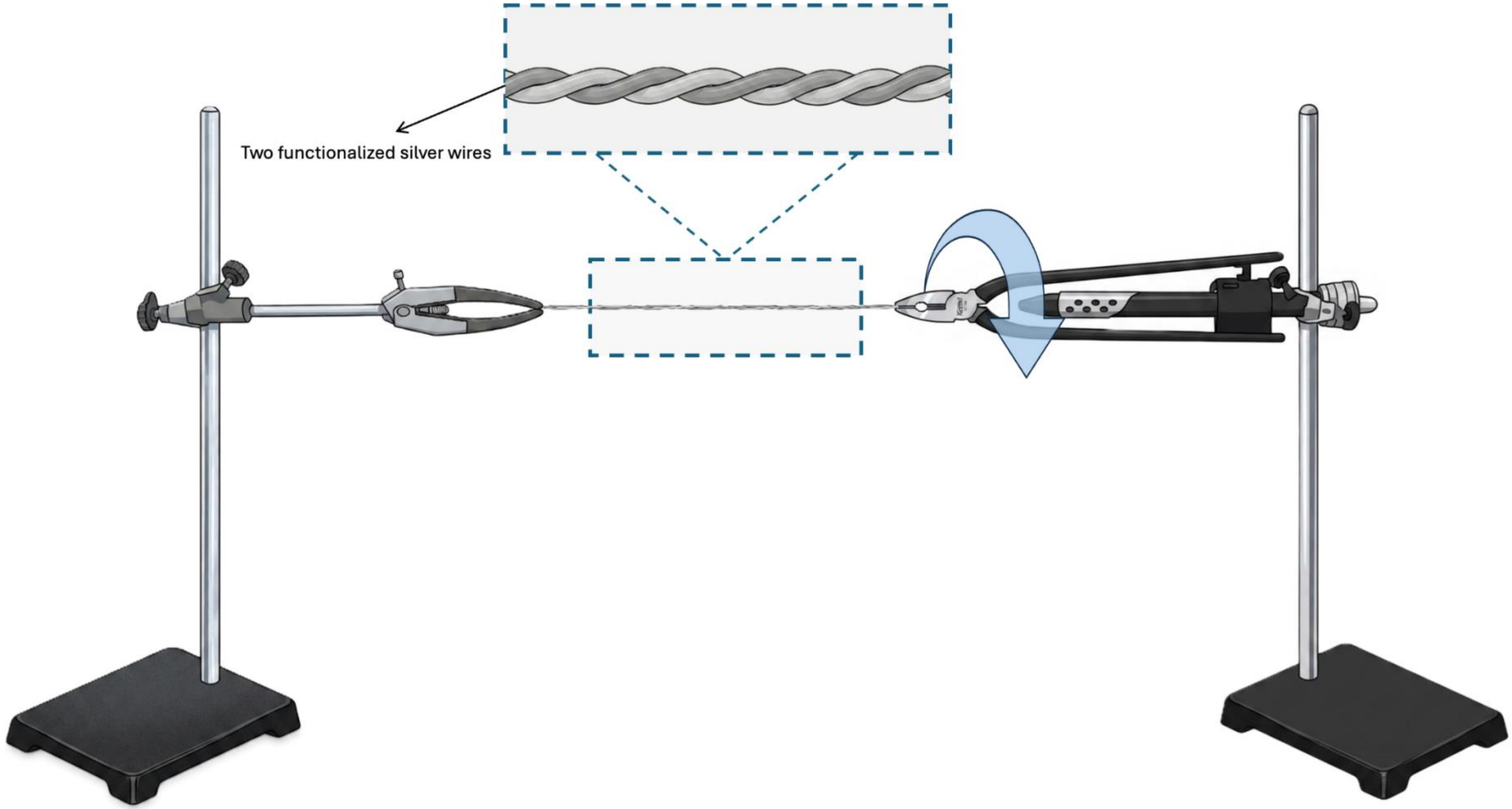

Figure S2. A) SEM images obtained of the CIM-80(*Al)* coating (top image) onto the SPME fibers and morphology of the crystallites (bottom image). **B)** FTIR spectra obtained with CIM-80(*Al)* MOF powders and with the MOF-based SPME fiber. **C**) PXRD patterns of CIM-80(*Al*) supported on fiber, the powder obtained in the reactor when synthesizing *in situ* the fiber, and the theoretical pattern.

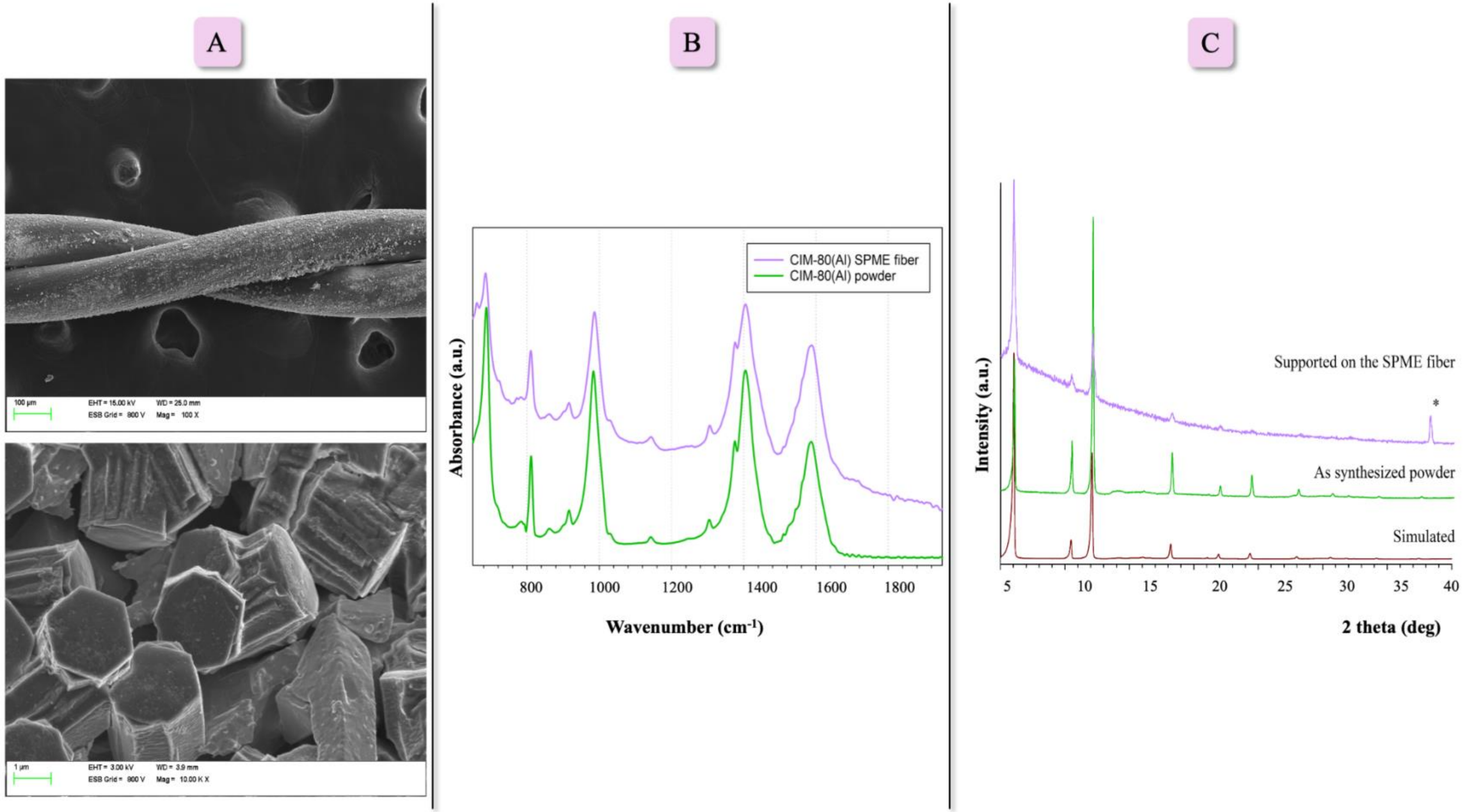

Figure S3. Results obtained when analyzing a PAHs standard at 10 µg·L^{-1} with four different MOF-coated braid fiber using HS-SPME under optimum conditions (57 ºC, 53 min with 20 % NaCl (*w/v*)). All experiments were performed by triplicate.

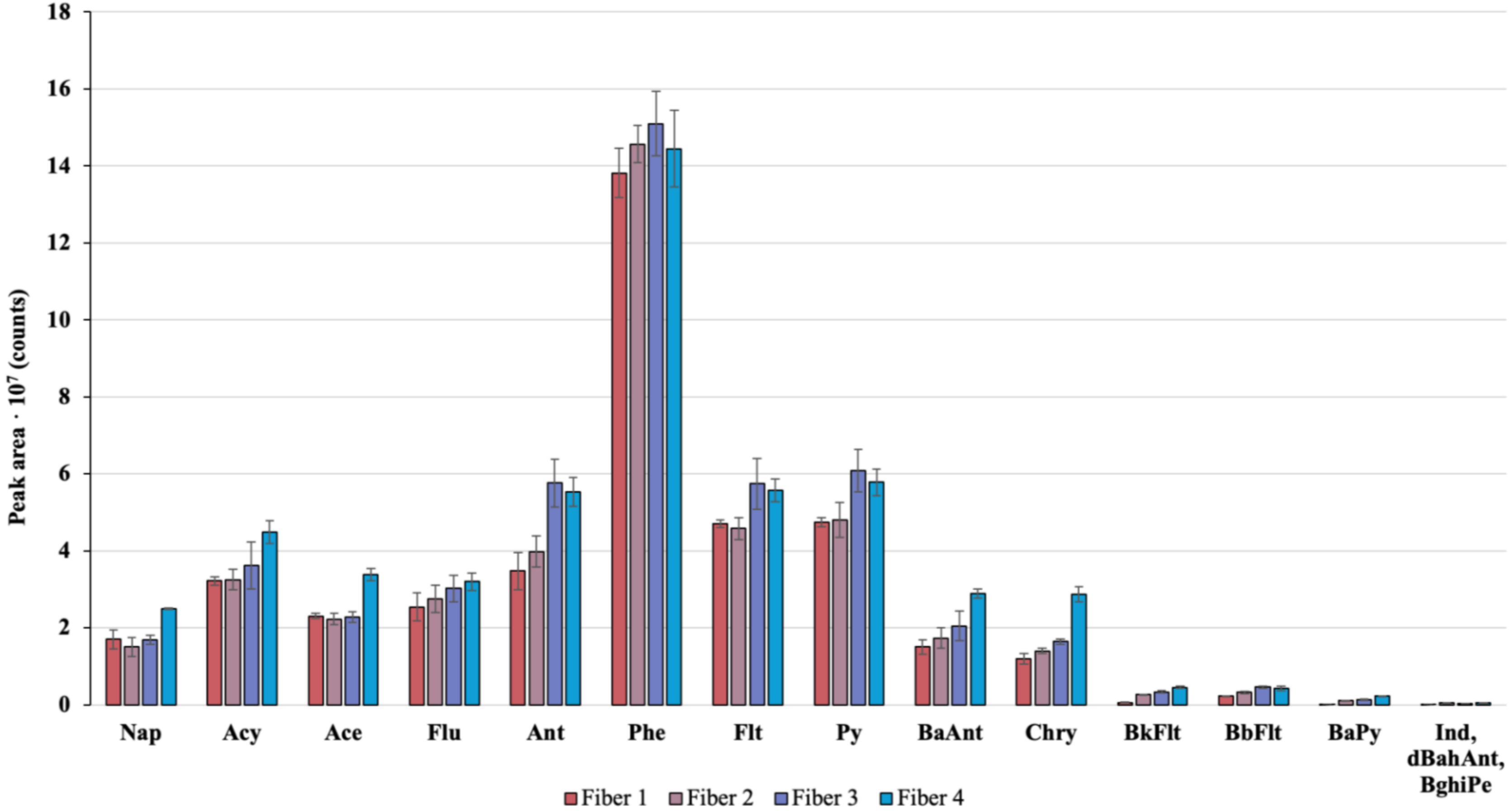

Figure S4. Surface responses and most favorable conditions for the entire group of target analytes: **A)** at 20% of NaCl content (*w/v*); **B)** using 60 °C for the extraction temperature; and **C)** using 60 min of extraction time.

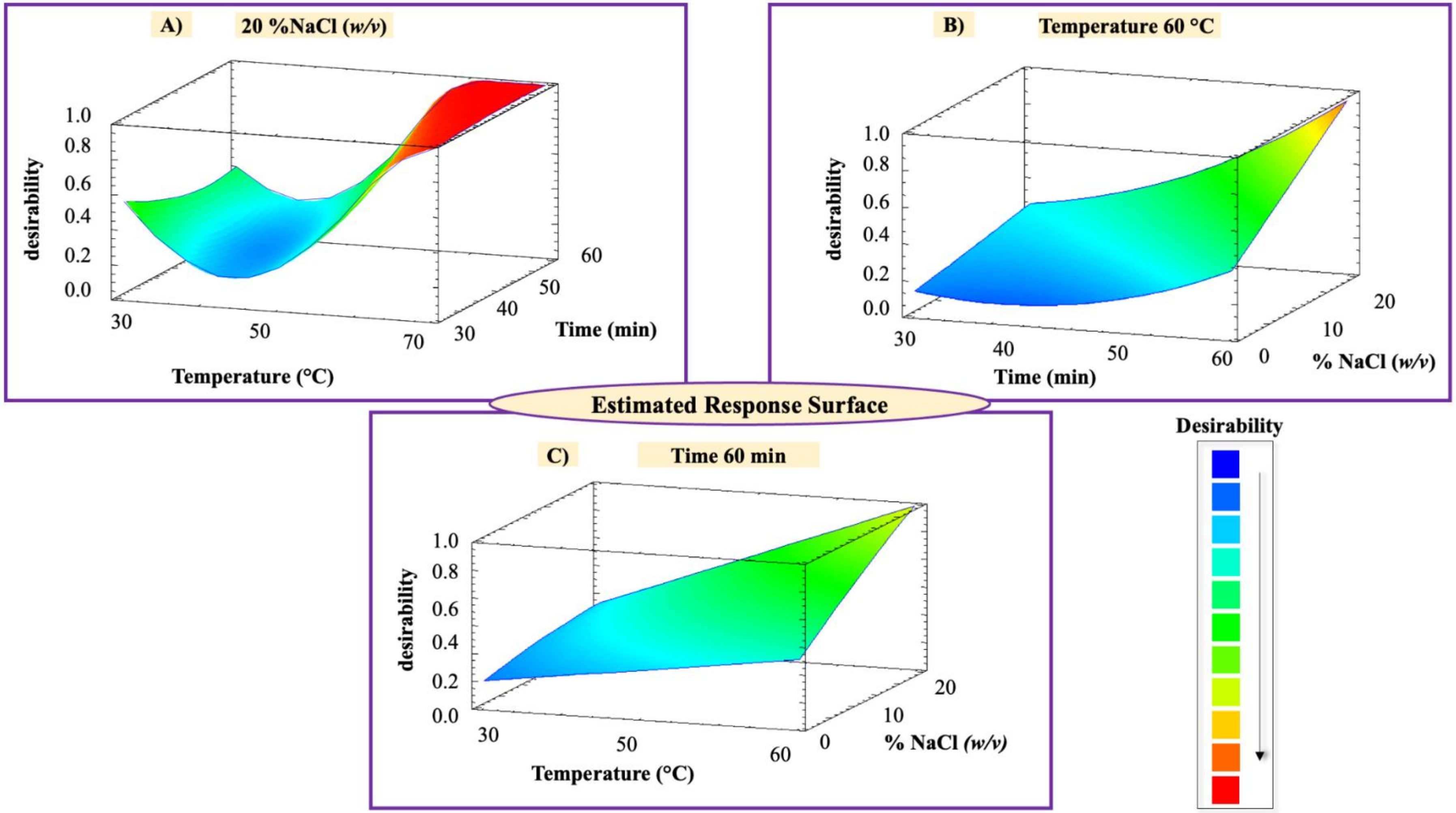

Figure S5. A) HS-SPME-GC-MS results, monitored as peak areas the VOCs group, using CIM-80(*Al)* with the optimum conditions of the commercial fibers, namely: condition 1) 62 °C, 60 min and 15% NaCl and condition; 2) 60 °C, 60 min and 20% NaCl, using 10 min 280 °C of temperature and desorption temperature in all cases, with experiments performed in triplicate. **B)** HS-SPME-GC-MS results, monitored as peak areas the VOCs group comparative between Car/PDMS under optimum conditions and CIM-80 (*Al*) under condition 2**.**

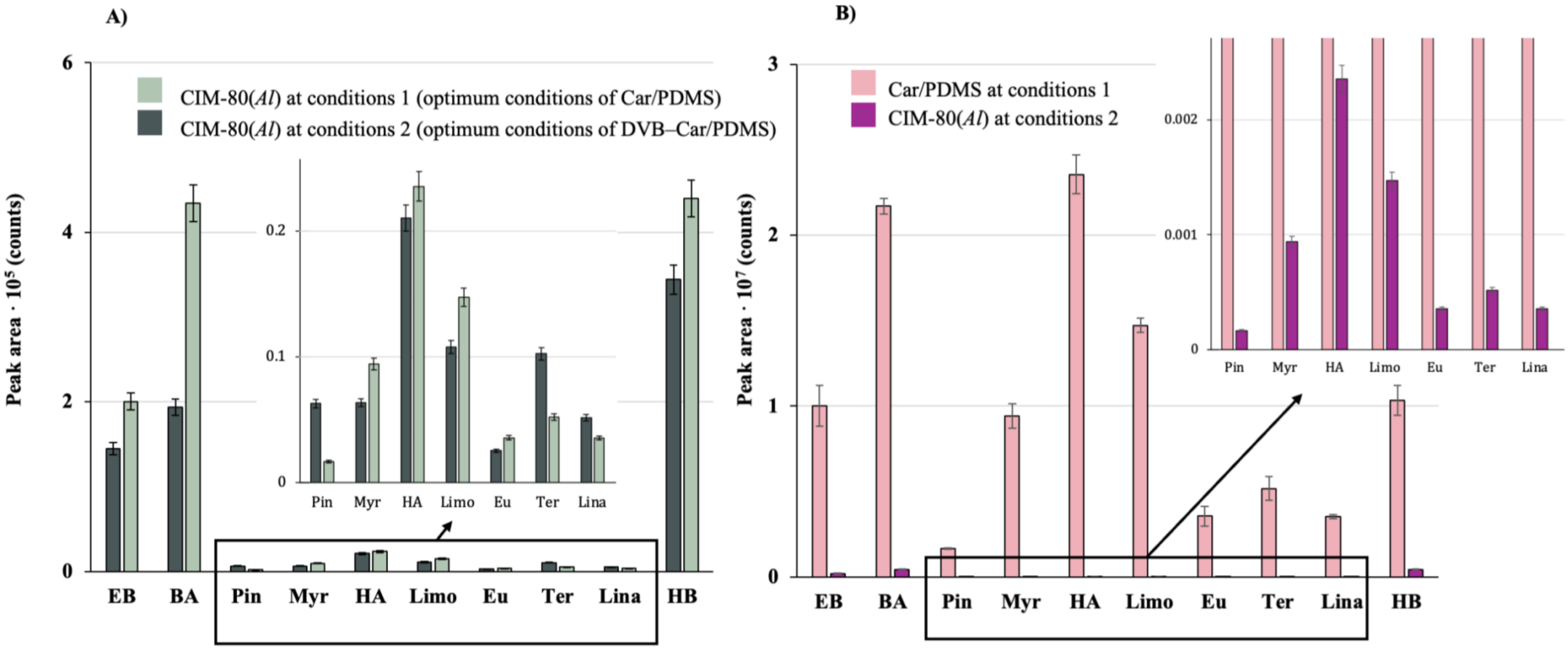

Figure S6. Families of compounds identified by each fiber in each SPME mode used (in white: the number of compounds identified that belongs to each family).

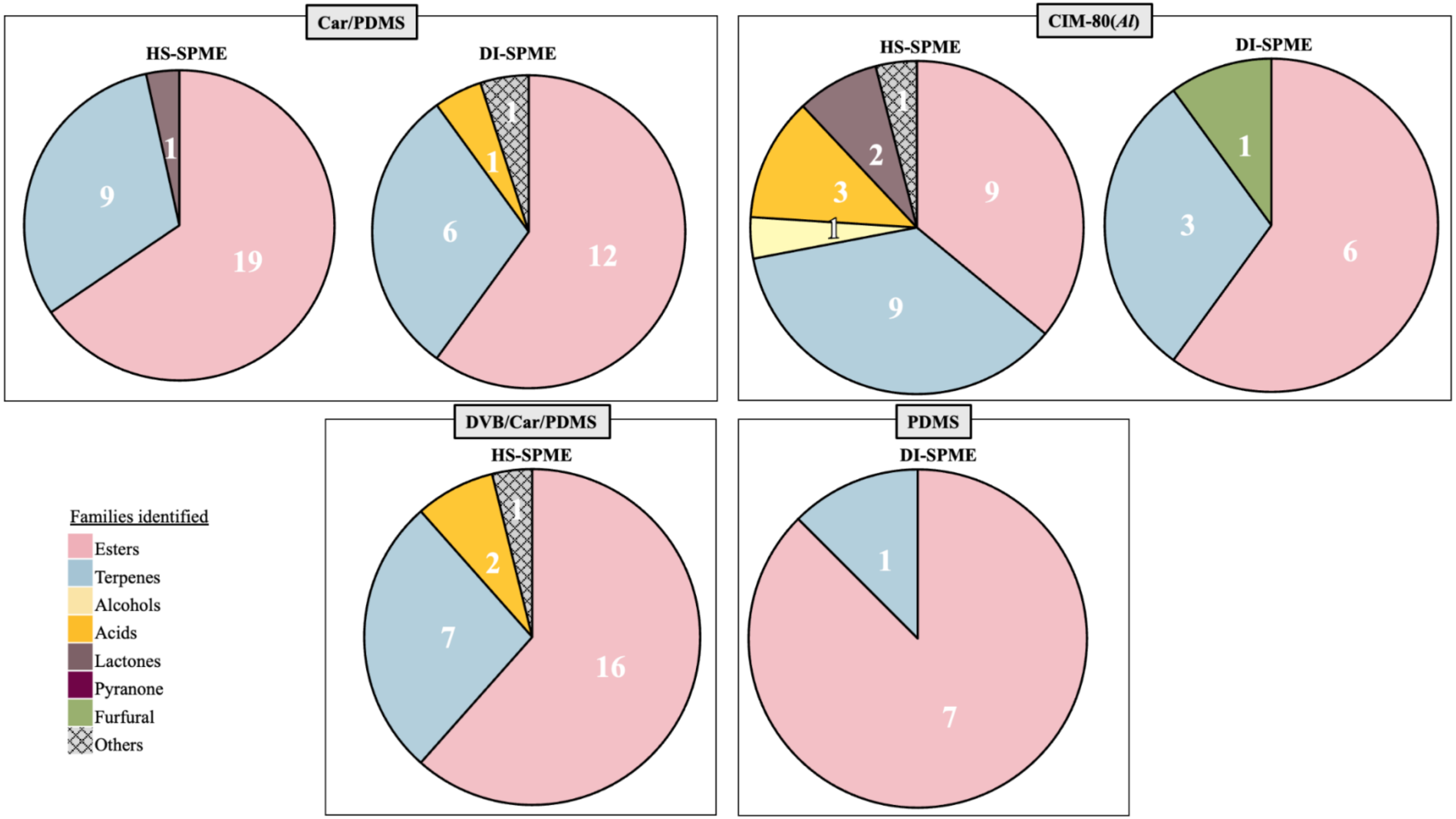

Figure S7. Results obtained after applying the HS-SPME-GC-MS method to passion fruit juice samples, with studies being performed in triplicate, while selecting the mass of the selected compounds in SIM mode. The analytes are shown in retention time order from left to right.

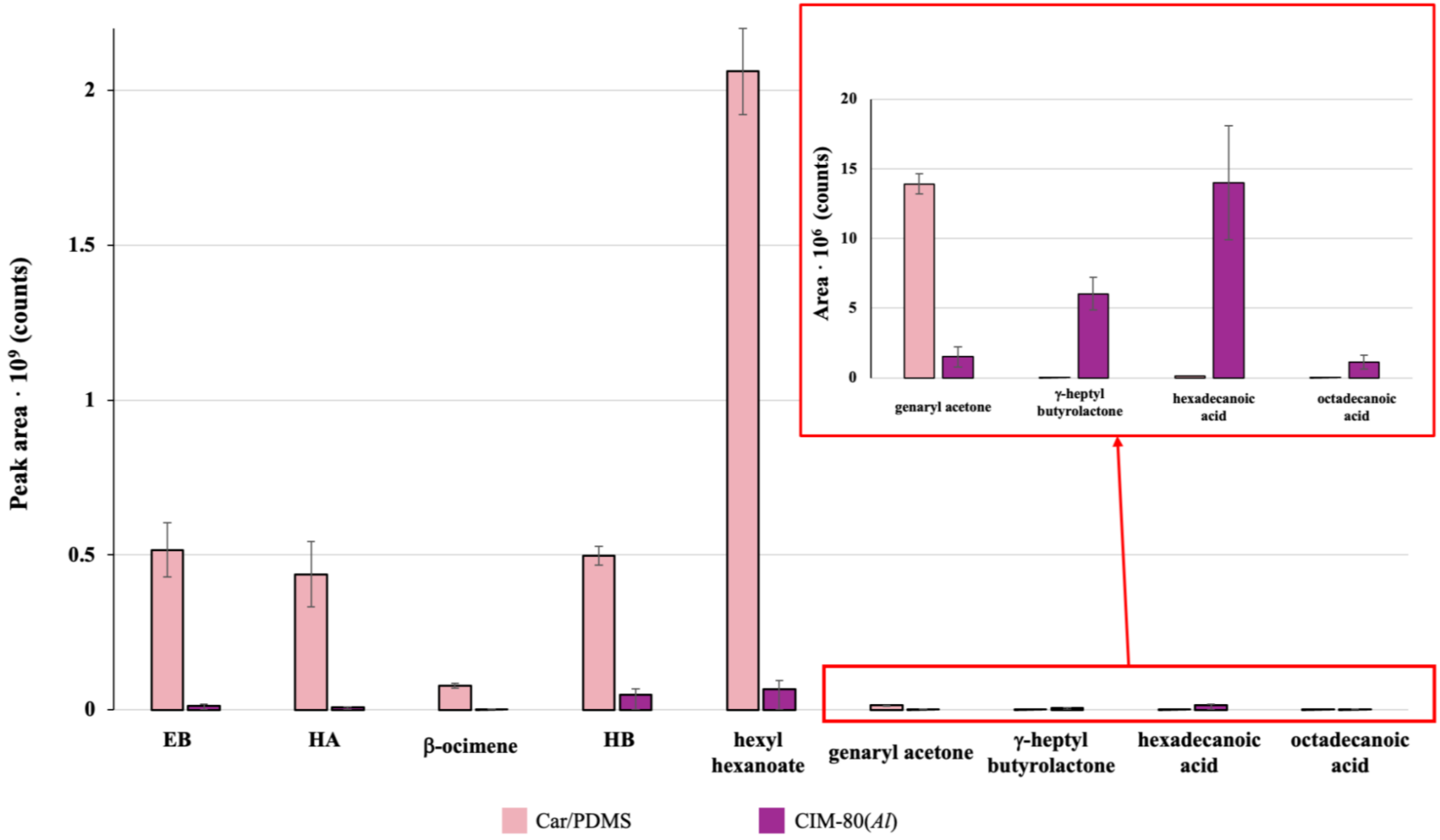

Figure S8. Results obtained after applying the DI-SPME-GC-MS method to passion fruit juice samples, with studies being performed in triplicate, while selecting the mass of the selected compounds in SIM mode.

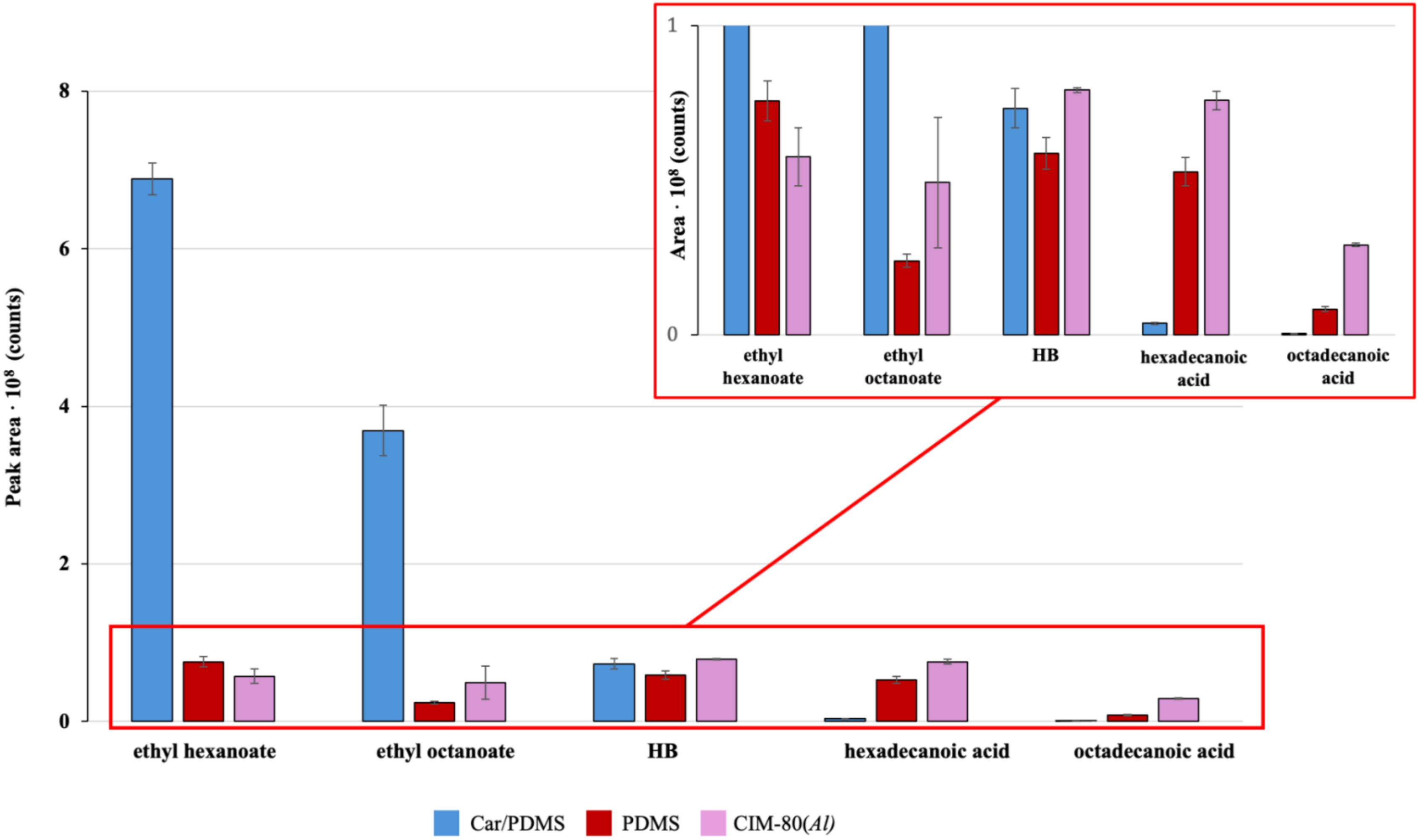

Figure S9. Results obtained when analyzing a PAHs standard with the SPME fibers before (green) immersing the fiber in the fruit-juice and after (pink). The experiments comprised three stages: 1) initial HS-SPME experiences of the PAH standard at 10 μg·L^{-1} (using optimum conditions for each fiber), 2) DI-SPME of the juice sample (60 min extraction, different extraction temperatures depending on the fiber, see Table S2), and 3) again HS-SPME of the PAH standard at 10 μg·L^{-1}. All experiments were performed by triplicate.

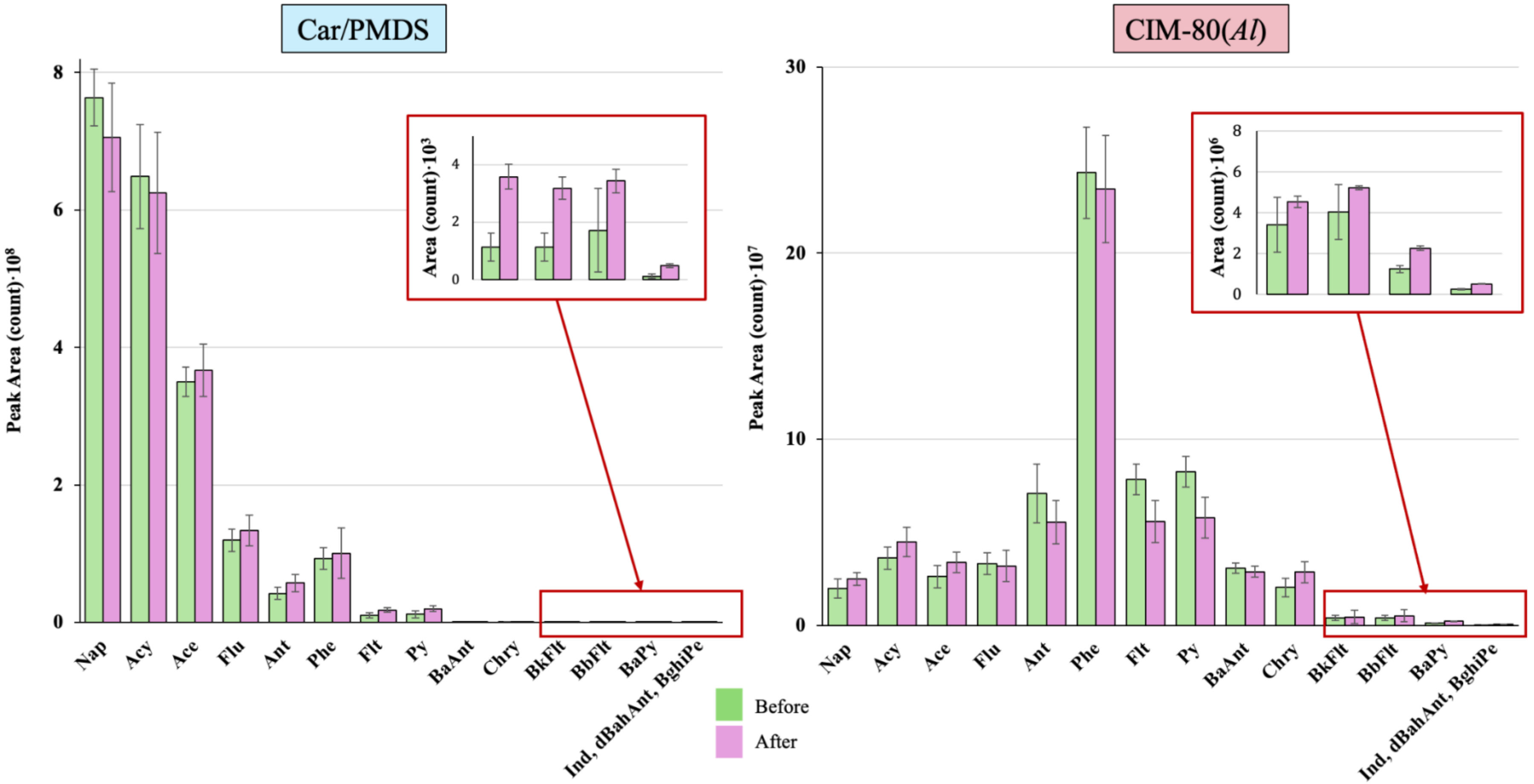

Table S1. Formula, chemical structure, and several physicochemical characteristics of the VOCs studied.

Analyte (abbreviation)	Formula	Chemical structure	Molecular weight ($g \cdot mol^{-1}$)	Boiling temperature (℃)	Vapor pressure (KPa) at 25 ℃
Ethyl butanoate (EB)	$C_6H_{12}O_2$	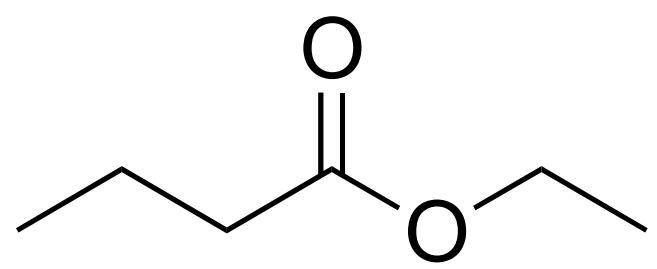	116.2	121	18.5
Butyl acetate (BA)	$C_6H_{12}O_2$	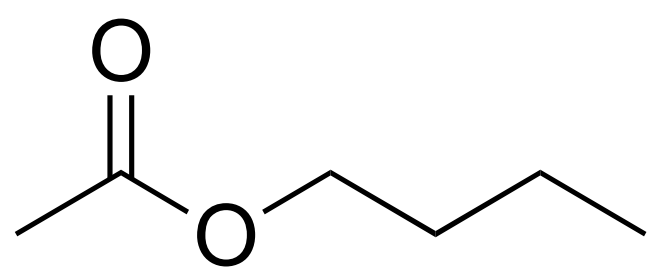	116.2	126	15.3
(−)-α-Pinene (Pin)	$C_{10}H_{16}$	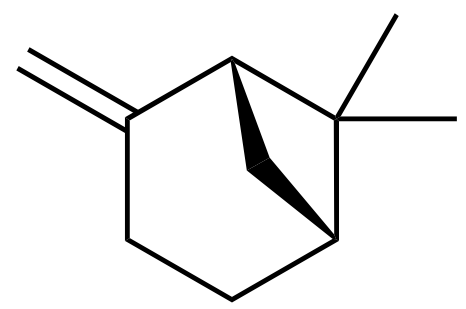	136.2	157	46.5
Myrcene (Myr)	$C_{10}H_{16}$	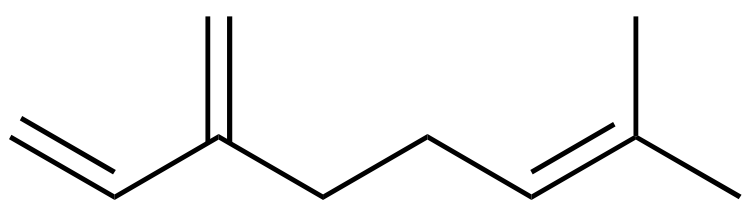	136.2	136	30.5
Hexyl acetate (HA)	$C_8H_{16}O_2$	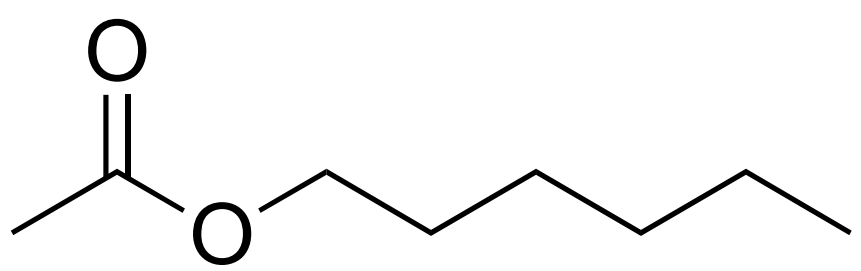	144.2	172	18.5

Table S1. Continued

Analyte (abbreviation)	Formula	Chemical structure	Molecular weight (g·mol^{-1})	Boiling temperature (°C)	Vapor pressure (KPa) at 25°C
(R)-(−)-Limonene (Limo)	$C_{10}H_{16}$	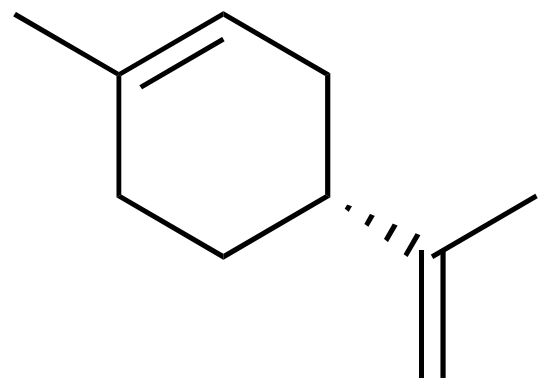	136.2	175	20.5
Eucalyptol (Eu)	$C_6H_{12}O_2$	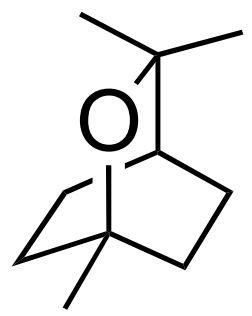	116.2	126	22.0
γ-Terpinene (Ter)	$C_{10}H_{16}$	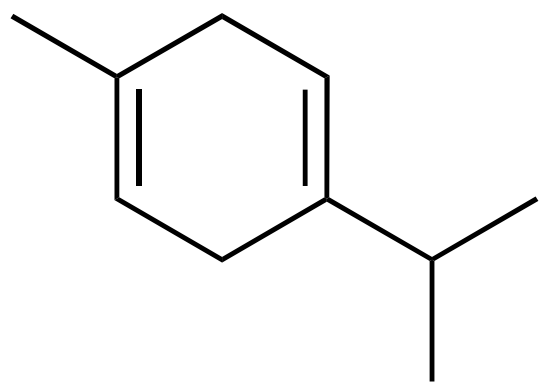	136.2	183	14.4
Linalool (Lina)	$C_{10}H_{18}O$	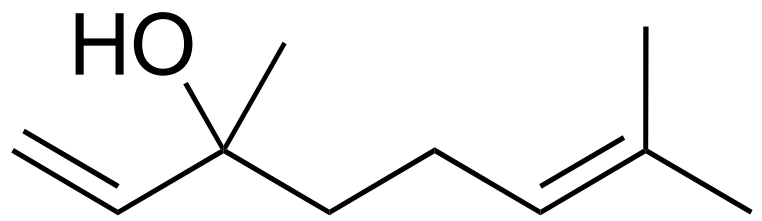	154.3	199	12.1
Hexyl butanoate (HB)	$C_{10}H_{20}O_2$	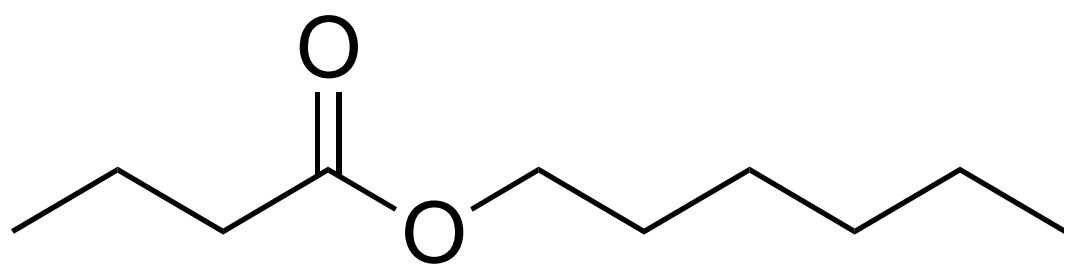	172.3	208	31.1

Note: Data from SciFinder Scholar© (American Chemical Society 2021).

Table S2. Summary of the extraction conditions used in this study for different experiments in combination with GC-MS.

**Extraction conditions**[1]	**Targeted/ untargeted analysis of passion fruit juice**			**PAH monitoring for assessing fiber lifetime after DI-SPME**
	**HS-SPME**	**HS-SPME**	**DI-SPME**	**HS-SPME**
Fibers	Car/PDMS[2]	DVB/Car/PDMS[2]<br>CIM-80(*Al*)[3]<br>PDMS[4]	Car/PDMS[4]<br>PDMS[4]<br>CIM-80(*Al*) [4]	Car/PDMS<br>PDMS<br>CIM-80(*Al*)
Sample volume (mL)	5	5	8	5
Vial capacity (mL)	10	10	10	10
NaCl content (%, w/v)	15	20	0	20
Extraction time (min)	60	60	60	57
Extraction temperature (ºC)	62	60	62	53
Stirring rate (rpm)	500	500	500	500

[1]Desorption conditions for all experiments:
Desorption time: 10 min; Desorption temperature: 270 ºC for DVB/Car/PDMS, 280 ºC for PDMS and for the MOF-based fiber, and 310 ºC for Car/PDMS.
[2]Optimum conditions studied via Doehlert design and/or previous studies.
[3]Optimum conditions selected in accordance with section 3.3.
[4]Conditions selected in accordance with section 3.3.
[4]Optimum conditions selected in accordance with section 3.4.

Table S3. Results of the extraction of a aqueous standard containing 16 PAHs using HS-SPME-GC-MS under optimal conditions (57 °C, 60 minutes, 20% (*w/v*) NaCl) with CIM-80(*Al*)-coated braid fiber.

**Compound**	**Fiber 1 RSD (%)[a]**	**Fiber 2 RSD (%)[a]**	**Fiber 3 RSD (%)[a]**	**Fiber 4 RSD (%)[a]**	**Inter-batch RSD (%)[b]**
Nap	14.6	13.3	7.2	1.1	13.6
Acy	3.2	8.3	17.0	6.7	11.8
Ace	2.9	6.5	6.3	4.5	4.3
Flu	14.7	12.8	11.4	7.1	13.7
Ant	14.1	10.3	10.8	6.6	18.4
Phe	4.6	3.3	5.5	6.8	5.5
Flt	2.0	6.4	11.6	5.2	12.1
Py	2.6	9.5	9.1	6.0	13.5
BaAnt	12.9	12.5	13.4	4.1	13.0
Chry	11.3	5.2	4.2	6.9	16.4
BkFlt	4.1	2.1	9.6	6.2	15.0
BbFlt	8.1	8.9	6.6	14.0	17.4
BaPy	2.7	6.4	15.1	4.8	18.2
Ind, dBahAnt, BghiPe	4.1	10.5	13.8	7.6	14.6

[a]Evaluated with $n = 3$ in the same day, using an aqueous standard at 10 $\mu g \cdot L^{-1}$
[b]Evaluated with $n = 9$ in different days and with different fibers, using an aqueous standard at 10 $\mu g \cdot L^{-1}$

Table S4. Coded values and operational levels of the Doehlert design used during the optimization of the HS-SPME-GC-MS (SIM mode) conditions with DVB/Car/PDMS.

**Experiment**	**Extraction temperature (°C)**		**Extraction time (min)**		**NaCl content (%, *w/v*)**	
	C_{Texp}	T_{exp}	C_{texp}	t_{exp}	C_{NaCl}	NaCl
1	0.00	50	0.00	45	0.00	12.5
2	1.00	70	0.00	45	0.00	12.5
3	0.50	60	0.87	60	0.00	12.5
4	0.50	60	0.29	50	0.82	25.0
5	-1.00	30	0.00	45	0.00	12.5
6	-0.50	40	-0.87	30	0.00	12.5
7	-0.50	40	-0.29	40	-0.82	0
8	0.50	60	-0.87	30	0.00	12.5
9	0.50	60	-0.29	40	-0.82	0
10	-0.50	40	0.87	60	0.00	12.5
11	0.00	50	0.58	55	-0.82	0
12	-0.50	40	0.29	50	0.82	25.0
13	0.00	50	-0.58	35	0.82	25.0
14	0.00	50	0.00	45	0.00	12.5
15	0.00	50	0.00	45	0.00	12.5

Note: C_i are the coded values for each factor level i. t_{exp}, T_{exp}, and NaCl are the real experimental conditions according to the following Equation:

$$R = \text{constant} + A[T_{ext}] + B[t_{ext}] + C[NaCl] + AA[T_{ext}]^2 + AB[T_{ext}][t_{ext}] + AC[T_{ext}][NaCl] + BB[t_{ext}]^2 + BC[t_{ext}][NaCl] + CC[NaCl]^2$$

Table S5. Constants and coefficients of the second order polynomial regression obtained for the response surfaces fitted for the target VOCs. Note: Doehlert design for HS-SPME optimization with DVB/Car/PDMS.

**Analyte**	**Constant (×10[7])**	**Coefficient[a] (×10[3])**								
		**A**	**B**	**C**	**AA**	**AB**	**AC**	**BB**	**BC**	**CC**
EB	1.01	-255	-211	170	0.96	4.14	-0.82	0.45	-1.68	0.62
BA	0.20	-66	-26	131	-0.22	1.90	-0.10	-0.43	-1.62	-0.25
Pin	-2.58	773	536	-178	-4.20	-4.01	-0.61	-5.02	8.68	-8.33
Myr	-1.17	487	-33	697	-5.19	7.09	-11.70	-2.79	1.99	-11.6
HA	2.90	-1343	113	1107	6.29	19.55	-8.93	-9.44	0.25	-20.7
Limo	3.36	-890	-167	-183	9.14	6.73	-6.86	-3.69	15.7	-12.3
Eu	-2.70	434	852	23	-4.56	-2.63	13.2	-5.75	-5.98	-7.40
Ter	0.72	-130	427	-203	9.06	-8.73	1.57	-2.35	6.76	-13.0
Lina	0.25	-644	529	295	2.71	8.24	3.39	-8.26	-0.63	-3.85
HB	5.18	-1210	-439	-405	10.7	7.80	1.91	-0.25	11.3	-14.8

[a] **A**: Extraction temperature (°C) coefficients, **B**: Extraction time (min) coefficients, and **C**: % NaCl (*w/v*) coefficients. **AA**, **BB**, and **CC** indicate quadratic effects, and **AB**, **AC**, and **BC**, indicate the interactions among variables. The response surface Equation is:

$$R = constant + A[T_{ext}] + B[t_{ext}] + C[NaCl] + AA[T_{ext}]^2 + AB[T_{ext}][t_{ext}] + AC\,[T_{ext}][NaCl] + BB[t_{ext}]^2 + BC[t_{ext}][NaCl] + CC[NaCl]^2$$

Table S6. Compounds identified in passion fruit juice using the HS-SPME-GC-MS method.

**Compound**	**RT[a] (min)**	**R Match**	**LRI_{exp}[b]**	**LRI_{lit}[c]**	**Car/ PDMS**	**DVB/Car/ PDMS**	**CIM-80(*Al*)**
ethyl butanoate (EB)	4.51	64.8	793	802	✓	✓	✓
ethyl-2-butenoate	5.26	80.8	800	834		✓	
β–pinene (Pin)	7.83	86.4	890	921	✓		
ethyl hexanoate	8.00	88.5	979	996	✓	✓	✓
hexyl acetate (HA)	8.21	79.3	992	953	✓		✓
o-cymene	8.45	78.9	1002	1015	✓		
1,5-dimethyl-6-methylenespiro[2.4]heptane	8.49	86.4	1008	1030			✓
limonene (Limo)	8.49	82.3	1008	1027	✓	✓	
benzyl alcohol	8.6.0	89.9	1010	1023			✓
ethyl-2-hexenoate	8.73	74.8	1020	1030	✓	✓	
β–ocimene	8.79	77.1	1030	1035	✓		✓
γ–terpinene (Ter)	8.99	78.7	1053	1062			✓
2-methyl-1-propenyl-benzene	9.51	93.2	1088	NF	✓		
propyl hexanoate	9.52	78	1089	1091		✓	
4,8-dimethylnona-1,3,7-triene	9.87	82.4	1091	1089		✓	
methyl octanoate	9.98	81	1099	1120		✓	
alloocimene	10.3	95.9	1120	1128	✓	✓	✓
octanoic acid	10.7	67.3	1160	1156			✓
4-hexen-1-yl butanoate	11.0	86.3	1164	1197	✓	✓	✓
3-hexen-1-yl butanoate	11.0	87.9	1165	1186	✓	✓	✓
hexyl butanoate (HB)	11.1	90.8	1170	1176	✓		✓
ethyl octanoate	11.2	86.8	1177	1181	✓		
octyl acetate	11.4	90.9	1180	1189		✓	
1-methylhexyl butanoate	11.5	82.2	1188	1193	✓		

Table S6. (Continued).

**Compound**	**RT[a] (min)**	**R Match**	**LRI_{exp}[b]**	**LRI_{lit}[c]**	**Car/ PDMS**	**DVB/Car/ PDMS**	**CIM-80(*Al*)**
butyl 2-butenoate	11.9	73.4	1120	1099			✓
ethyl 2-octenoate	12.0	66.4	1221	1243	✓		
theaspirane	13.4	86.7	1303	1302	✓	✓	
1,5,5-trimethyl-6-methylene-cyclohexene	13.7	80.8	1310	1338			✓
phenylmethyl butanoate	14.0	81.8	1320	1312	✓		
3-hexenyl hexanoate	14.6	90.5	1361	1371	✓		
hexyl hexanoate	14.7	71.8	1364	1371	✓	✓	✓
heptyl hexanoate	15.0	87.8	1430	1448		✓	
(Z)-geranylacetone	15.7	86.7	1452	1455	✓	✓	✓
butyl 2-hexenoate	15.7	83.5	1436	NF		✓	
dihydro-β-ionone	15.9	84.8	1437	1436	✓	✓	
3,3-dimethyl-4-phenyl-4-penten-2-one	16.0	81.8	1440	NF			✓
ethyl cinnamate	16.3	93.2	1460	1464		✓	
γ–heptyl butyrolactone	16.4	83.3	1469	1470			✓
β–ionone	16.8	70.4	1472	1486		✓	✓
dihydroactinidiolide	17.8	63.6	1495	1498	✓	✓	✓
4-methyl-phenylmethyl pentanoate	18.0	79.1	1499	1493	✓		
geranyl isobutyrate	18.3	72.5	1500	1511	✓		
3-hexenyl octanoate	18.7	77.9	1542	1562	✓		
octyl hexanoate	18.8	81.6	1542	1570	✓		
pseudoionone	18.8	70.1	1543	1527			✓
3-mercaptohexyl hexanoate	19.7	73.8	1589	1621	✓		✓
γ–dodecalactone	20.8	77.8	1599	1582			✓
citronellyl isohexanoate	21.6	75.5	1680	1687		✓	

Table S6. (Continued).

Compound	RT[a] (min)	R Match	LRI_{exp}[b]	LRI_{lit}[c]	Car/ PDMS	DVB/Car/ PDMS	CIM-80(*Al*)
octyl octanoate	22.8	88.5	1750	1767		✓	
isopropyl myristate	23.8	81.3	1802	1814	✓	✓	
hexadecanoic acid	26.3	88.2	1845	1848		✓	✓
octadecanoic acid	30.0	77.9	2136	2167		✓	✓

[a] Retention time
[b] Experimental Linear Retention Index
[c] Literature Linear Retention Index (search in NIST Chemistry WebBook)

Table S7. Comparison of analytical methods developed for the analysis of passion fruit via SPME-GC.

Reference	Goal of the study	Sample / analytical method	Number of VOCs	Comments, comparison with respect of this study
Studies focused on new SPME coatings or new sample preparation				
This study	To demonstrate the complementary nature of the developed MOF fibers with commercial coatings for untargeted analysis of passion fruit juices	pool of passion fruit juice / MOF fiber, DVB/Car/PDMS, Car/PDMS, and PDMS, HS- and DI-SPME-GC-MS	53	This study did not analyze different juices independently but combined all juices to create a pool (thus to avoid the variability between samples) while exploring the possibility of using a MOF fiber as complementary to commercial ones to ensure a more holistic passion fruit analysis
[28]	To study the extraction efficiency of organophosphorus pesticides with MOF-coated fibers	water samples and orange, apple, and grape juices / PAN/Ni-MOF fiber, HS-SPME-CD-IMS	2	This study performed a quantitative analysis of two target compounds
[29]	To study the strategy for detecting trace of polychlorinated biphenyls in complex samples with MOF-coated fibers	lake water, mineral water and orange juice / MIL-on-UiO fiber, HS-SPME-GC-FID	5	This study performed a quantitative analysis of five target compounds
[30]	To study the extraction efficacy of organophosphorus pesticides with a synthesis MOF-coated fibers	apple, potato, grape juice, tomato and river water / GNP/Al-MOF fiber, HS-SPME-GC-MS	4	This study performed a quantitative analysis of four target compounds

[31]	To study the extraction efficiency of triazole fungicides with a MOF-coated fiber	grape, apple, cucumber, celery cabbage, pear, cabbage, and tomato samples/ MOF-5/GO fiber, DI-SPME-GC-μECD	5	This study performed a quantitative analysis of five target compounds
[32]	To study two approaches for the determination of volatile and semi-volatile PAHs.	apple, pear, plum and grapefruit/ Car/PDMS, HS-SPME-GC for light compounds	11	This study performed a quantitative analysis of eleven target compounds
Studies focused on elucidating the volatile profile of different passion fruit varieties				
[11]	To study the differences in the volatile profile of different passion fruit varieties and to apply multivariate analysis to discriminate between vegetative species	passion fruit juices from 9 plant varieties / DVB/Car/PDMS-HS-SPME-GC-qMS	169	Higher number of VOCs were detected as different and independent samples were analyzed
[12]	To study the differences in the volatile profile of different passion fruit varieties and to apply linear discrimination analysis to discriminate between vegetative species	18 juice samples obtained from 3 varieties of passion fruit / Car/PDMS-HS-SPME-GC-QqQ MS	135	Higher number of VOCs than as samples were analyzed independently
Studies focused on elucidating the volatile profile after injury, infection, or different juice post-processes				
[1]	To study the effect of fermentation in the volatile profile of passion fruit wine	passion fruit wine (obtained from passion fruit juice fermentation) / DVB/Car/PDMS-HS-SPME / Either GC-Orbitrap-MS or GC-qMS	17 VOCs by GC-Orbitrap-MS, and 78 VOCs by GC-MS	Similar results to the current study in terms of number of VOCs
[13]	To study the effect of ripening in the volatile profile of passion fruit juice	passion fruit juice (juices from different ripening stages) / DVB/Car/PDMS-HS-SPME-GC-qMS	31	Identification by library NIST and RIs. Similar results to the current study in terms of types of VOCs identified

[14]	To study the effect of post-harvest ripening processes in the volatile profile of passion fruit juice	3 varieties of passion fruit and 3 processes / DVB/Car/PDMS-HS-SPME-GC-MS	605	VOC identification was based on the identification of 3 characteristics m/z ions via SIM; RIs are not reported
[15]	To study the effect of a pasteurization process (i.e., dielectric barrier discharge cold plasma) in the volatile profile of passion fruit juice	passion fruit juice (fresh and subjected to cold plasma) / DVB/Car/PDMS-HS-SPME-GC-qMS	70	Similar results than the current study in terms of number of VOCs
[16]	To study the effect of pasteurization processes (i.e., thermal procedures) in the volatile profile of passion fruit juice	passion fruit juice (fresh and subjected thermal treatments) / DVB/Car/PDMS-HS-SPME-GC-qMS	63	Similar results than the current study in terms of number of VOCs
[17]	To study the effect of a pasteurization process (i.e., a thermal procedure) in the volatile profile of passion fruit juice	passion fruit juice (fresh and subjected thermal treatment) / DVB/Car/PDMS-HS-SPME-GC × GC-TOF MS	134 VOCs in fresh juice, and 102 VOCs in processed juice	Higher number of VOCs than the current study as GC × GC-TOF MS is more powerful and sensitive
[42]	To monitor the change of the volatile profile of passion fruit juices after virus infection	passion fruit juice (fresh and after virus injection) / DVB/Car/PDMS-HS-SPME-GC-QqQ MS	559 VOCs	The data processing and identification protocol was based on SIM confirmation. There is a report of theoretical retention indexes, but absence of experimental retention indexes

Table S8. The m/z values used for the selected compounds in HS-SPME mode.

Analyte	Retention time (min)	Quantifier (m/z)	Qualifier (m/z)
ethyl butanoate (EB)	4.51	71	43, 88
hexyl acetate (HA)	8.21	43	55, 56, 61
β-ocimene	8.79	93	41, 79, 77
hexyl butanoate (HB)	11.1	89	43, 56, 71
hexyl hexanoate	14.7	43	55, 69, 41
(Z)-geranylacetone	15.7	43	69, 41, 107
γ-heptyl butyrolactone	16.4	85	128, 41, 55
hexadecanoic acid	26.3	43	73, 60,57
octadecanoic acid	30.0	73	60, 43, 57

Table S9. Compounds identified in passion fruit juice using the DI-SPME-GC-MS method.

Compound	RT[a] (min)	R Match	LRI_{exp}[b]	LRI_{lit}[c]	Car/ PDMS	PDMS	CIM-80(*Al*)
ethyl butanoate (EB)	4.51	72.6	793	802	✓		
5-methylfurfural	7.42	71.5	949	963			✓
ethyl hexanoate	7.98	70.1	979	996	✓	✓	✓
hexyl acetate (HA)	8.21	62.9	992	953	✓		
limonene (Limo)	8.49	67.8	1008	1027	✓		
1-octanol	9.16	84.4	1053	1065	✓		
4,8-dimethyl-1,3,7-nonatriene	9.89	68.7	1097	1110	✓		
ethyl-3-hydroxy-hexanoate	10.1	59.1	1120	1133	✓		
3,4-dimethyl-2,4,6-octatriene	10.3	89.8	1122	1132	✓		
3-hexen-1-yl butanoate	11.0	84.6	1165	1185	✓		
hexyl butanoate (HB)	11.1	78.2	1170	1176	✓	✓	✓
ethyl octanoate	11.2	67.7	1176	1181	✓	✓	✓
1-methylhexyl butanoate	11.5	80.5	1188	1194	✓		
hexyl-2-butenoate	12.0	74.9	1221	NF	✓		
theaspirane	13.4	81.8	1303	1302	✓		
3-hexenyl hexanoate	14.6	77.5	1361	1381	✓	✓	✓
hexyl hexanoate	14.7	89.9	1364	1371	✓	✓	✓
(Z)-geranylacetone	15.7	74.8	1452	1455	✓		
β–ionone	16.8	89.2	1472	1486	✓		
dihydroactinidioide	17.8	66	1495	1498	✓		
hexyl octanoate	18.7	68.5	1562	1581	✓	✓	✓
octyl hexanoate	18.8	80.9	1572	1570		✓	

Table S9. (Continued).

**Compound**	**RT[a] (min)**	**R Match**	**LRI_{exp}[b]**	**LRI_{lit}[c]**	**Car/ PDMS**	**DVB/Car/ PDMS**	**CIM-80(*Al*)**
hexadecanoic acid	26.5	86.2	1845	1848			✓
9-hexadecenoic acid	28.7	72.8	1960	1942			✓
octadecanoic acid	30.1	79.1	2136	2167			✓
trans-geranylgeraniol	31.5	85.1	2206	2201		✓	

[a] Retention time
[b] Experimental Linear Retention Index
[c] Literature Linear Retention Index (search in NIST Chemistry WebBook)

Table S10. The m/z values used for the selected compounds in DI-SPME mode

Analyte	Retention time (min)	Quantifier (m/z)	Qualifier (m/z)
ethyl hexanoate	7.98	43	60, 88, 99
hexyl butanoate (HB)	11.1	89	43, 56, 71
hexyl octanoate	14.7	145	127, 84, 57
hexadecanoic acid	26.3	43	73, 60,57
octadecanoic acid	30.0	73	60, 43, 57
